# Recent advances in the treatment and delivery system of diabetic retinopathy

**DOI:** 10.3389/fendo.2024.1347864

**Published:** 2024-02-15

**Authors:** Zhiyi Wang, Ningzhi Zhang, Pei Lin, Yiqiao Xing, Ning Yang

**Affiliations:** Department of Ophthalmology, Renmin Hospital of Wuhan University, Wuhan, Hubei, China

**Keywords:** diabetic retinopathy, laser treatment, anti-VEGF drugs, gene therapy, drug-delivery system, nanotechnology

## Abstract

Diabetic retinopathy (DR) is a highly tissue-specific neurovascular complication of type 1 and type 2 diabetes mellitus and is among the leading causes of blindness worldwide. Pathophysiological changes in DR encompass neurodegeneration, inflammation, and oxidative stress. Current treatments for DR, including anti-vascular endothelial growth factor, steroids, laser photocoagulation, and vitrectomy have limitations and adverse reactions, necessitating the exploration of novel treatment strategies. This review aims to summarize the current pathophysiology, therapeutic approaches, and available drug-delivery methods for treating DR, and discuss their respective development potentials. Recent research indicates the efficacy of novel receptor inhibitors and agonists, such as aldose reductase inhibitors, angiotensin-converting enzyme inhibitors, peroxisome proliferator-activated receptor alpha agonists, and novel drugs in delaying DR. Furthermore, with continuous advancements in nanotechnology, a new form of drug delivery has been developed that can address certain limitations of clinical drug therapy, such as low solubility and poor penetration. This review serves as a theoretical foundation for future research on DR treatment. While highlighting promising therapeutic targets, it underscores the need for continuous exploration to enhance our understanding of DR pathogenesis. The limitations of current treatments and the potential for future advancements emphasize the importance of ongoing research in this field.

## 1 Introduction

Diabetic retinopathy (DR) is a serious complication of diabetes mellitus (DM) that affects almost 103.12 million people worldwide (1). Its prevalence is increasing with the aging global population. According to a 2020 meta-analysis, DR is one of the leading causes of irreversible blindness in people aged over 50 years (2). Between 1990 and 2015, the number of people blinded due to DR increased from 2 million to 4 million, and the number of people with visual impairment due to DR increased from 1.4 million to 2.6 million (3). DR continues to be a prominent cause of vision loss in many developed and developing countries, incurring a substantial socioeconomic cost to the healthcare system, with a considerable increase in healthcare costs with progressing illness from the early to late stages (4).

However, research on DR is still limited to microangiopathy, neovascularization, and neurodegeneration under hypoxic and inflammatory conditions. Further, its specific pathology and pathogenesis have not been fully elucidated. Panretinal photocoagulation is the first-line treatment to prevent visual loss in patients with DR, but the destructive nature of the laser can bring a series of ocular complications, such as: visual acuity, visual field damage. Other treatments have varying degrees of limitations and adverse effects, for example, intravitreal injections of anti-vascular endothelial growth factor (VEGF) drugs not only fail to completely inhibit the progression of DR, but frequent injections lead to an increased incidence of endophthalmitis (5). New therapies, such as novel laser photocoagulation combined with small incision vitrectomy and nanotherapy, are less destructive to the retina than older therapies, and may be useful in patients who respond poorly to conventional treatments (6). It is worth mentioning that nanotechnology and artificial intelligence have rapidly developed recently, providing new ideas in drug-delivery methods, early monitoring, and remote monitoring to treat DR (7–9).

In this review, we summarize the current pathophysiology, therapeutic approaches, and drug-delivery methods for treating DR, and discuss their respective development potentials.

## 2 Pathophysiology of DR

DR is a highly tissue-specific neurovascular complication of DM (10). In the retina, various cells communicate and collaborate to maintain a healthy retinal environment and function. Among them, neurons, glial cells, and blood vessels form the neurovascular unit (NVU), which gets subjected to primary pathological change in early DR (11).

According to international clinical grading criteria, DR can be divided into six stages belonging to two categories. The first category, non-proliferative diabetic retinopathy (NPDR), includes microangiomas and small blebs (stage 1), hard exudates (stage 2), and cotton-like soft exudates (stage 3). The second category, proliferative diabetic retinopathy (PDR), includes neovascularization and vitreous hemorrhage (stage 4), proliferation of fibrovascular and vitreous tissue (stage 5), and retinal detachment and stretching resulting in blindness (stage 6) (12).

**Figure 1:**
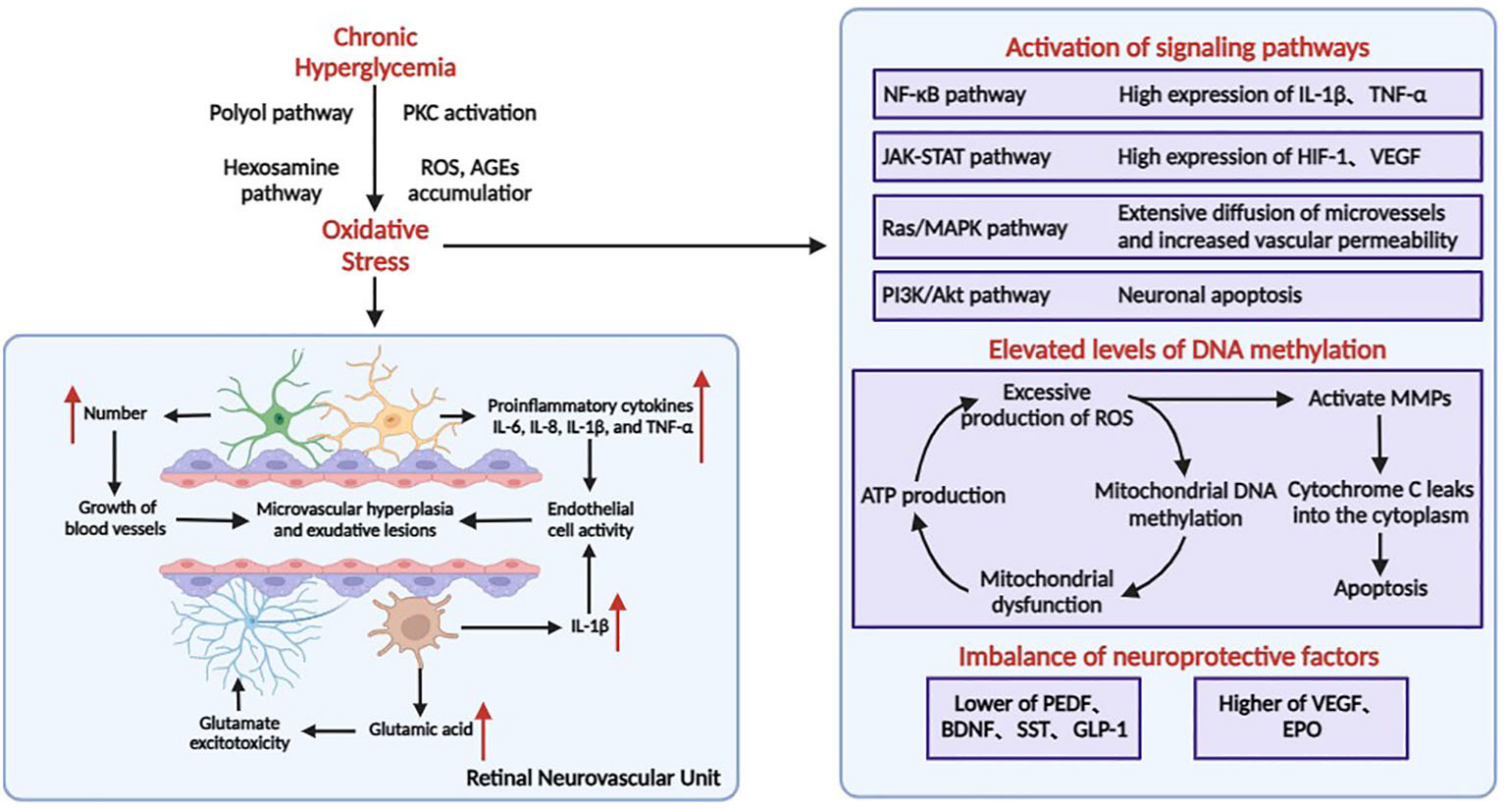
Pathophysiological process of DR. In the retina, neurons, Müller cells, astrocytes, microglia, endothelial cells, and pericytes join together to form neurovascular units. The pathology of DR is mainly characterized by high glucose–induced inflammatory response, leading to microangiopathy and neurodegenerative lesions.

NPDR is the initial stage and the most common form of DR. Hyperglycemia leads to increased vascular permeability and pericyte death (13). Loss of pericytes may further result in the destruction of endothelium-pericyte crosstalk and basement membrane thinning (14). Loss of pericytes and endothelial cells can lead to microaneurysms and capillary obstruction, resulting in retinal ischemia and hypoxia (15). Subsequently, elevated levels of intravitreal angiopoietin and VEGF induce pathological neovascularization, progressing to PDR (16) (**Figure 1**). PDR is characterized by the abnormal formation of new vessels on the retinal or optic disc surface and subsequent fibrosis. Owing to frequent hemorrhage from neovascularization, contractible fibrovascular retinal adventitia is formed, leading to serious complications such as tractional retinal detachment and blindness.

Loss of retinal ganglion cells (RGCs) and reduction in retinal thickness have been observed before the appearance of microangiopathy, indicating that DR is microangiopathy characterized by neurodegenerative degeneration (17). Neuronal cell death and axonal degeneration abnormalities are irreversible and directly related to vision loss in patients with diabetes. In the early and chronic stages of DR, elevated levels of various inflammatory cytokines and chemokines can be monitored in patients’ serum as well as in vitreous and atrial fluid (18). Many studies have shown that microglia in diabetic patients shift from an anti-inflammatory to a pro-inflammatory state and secrete pro-angiogenic factors (19). Consequently, the massive chronic inflammatory response exacerbates cell death and axonal degeneration of DR retinal neurons, which subsequently leads to vision loss in patients (20). Some neurotrophic factors, such as neurotrophin-4 (NT-4), brain-derived neurotrophic factor, taurodeoxycholic acid, and cytarabine, can exhibit neuroprotective and regenerative effects in the retina exposed to diabetic stress (21–24).

Elevated levels of DNA methylation have received increasing attention in the pathogenesis of DR (25). Chronic hyperglycemia can lead to the overproduction of intracellular advanced glycosylation end products (AGEs) and activation of protein kinase C (PKC), polyol, and hexosamine pathways, and disruption of antioxidant defenses all contribute to the production of large amounts of reactive oxygen species (ROS) in the retina, resulting in increased oxidative stress (26) (**Figure 1**). Mitochondria are both a major source of ROS and a major target site for ROS oxidative damage (27). Overproduction of ROS causes elevated methylation of retinal mitochondrial DNA (mtDNA), affects mitochondrial gene expression, impairs the function of the electron transport chain, leads to a decrease in the membrane potential of mitochondria resulting in mitochondrial dysfunction, and induces apoptosis, which contributes to the development of DR (27). At the same time, the impaired function of the electron transport chain affects ATP production, which further leads to an increase in free oxygen radicals (ROS), thus creating a “vicious circle”.

Clustered ROS affects the expression of the Bcl-2 family of mitochondrial apoptosis-related proteins. Increased ROS in the mitochondria or cytoplasm activates MMPs, a family of extracellular matrix-degrading proteins associated with angiogenesis, vascular remodeling, and apoptosis. MMPs are translocated from the cytoplasm to the mitochondria with the help of heat shock proteins (Hsp70), and impair the integrity of the mitochondria by regulating the expression of Hsp60 and gap junction proteins, leading to leakage of cytochrome C into the cytoplasm and initiating the apoptotic mechanism. As an important intracellular signaling molecule, excessive accumulation of ROS can also gradually activate the NF-κB and MAPK cascade response to promote the release of inflammatory factors, inducing an inflammatory response and further exacerbating the progression of DR disease.

While classic risk factors for DR (hyperglycemia, hypertension, dyslipidemia, and others) help stratify the possibility of a patient for progressing DR, numerous DM without these factors progress DR; additionally, there exist long-duration DM patients who progress DR by no means. The occurrence and progress of microvascular complications could be reduced by rigorous glycemic control to some extent, viz. hemoglobin A1c (HbA1c) holds some value for DR progression. However, HbA1c accounts for only 6.6% in the variation of the DR risk. Thus, the identification of novel and impactful biomarkers for DR remains urgent (28). A large number of research data suggest that in the control and treatment of DR, stabilization of the mitochondrial structure, inhibition of related inflammation, modification of target genes, and prevention of oxidative stress are feasible, and the complex interactions between multiple mechanisms deserve in-depth study (29). Mounting evidence demonstrates that inflammation is a key mechanism driving diabetes-associated retinal disturbance, yet the pathophysiological process and molecular mechanisms of inflammation underlying diabetic retinopathy are not fully understood. Cytokines, chemokines, and adhesion molecules interact with each other to form a complex molecular network that propagates the inflammatory and pathological cascade of diabetic retinopathy (30). Three types of molecules perform their own functions, while an increase in the level of one type of molecule also induces the expression of another type of molecule in an inflammatory state. In the future, the immune inflammation mechanism of DR can be further studied to provide insight into the biological function of DR and related targeted therapies.

## 3 Treatment of DR

### 3.1 Laser treatment

Laser photocoagulation has been widely used to prevent the progression from severe NPDR to PDR. Photocoagulation in non-perfused retinal areas reduces retinal neovascularization and lowers vascular growth factor and VEGF levels in retinal tissues (31). Clinical data have shown that laser treatment can considerably reduce the risk of severe vision loss in DR patients (32). However, the exact mechanism by which retinal laser treatment leads to effective treatment and improvement in retinal vascular disease is not fully understood.

Long-term studies have revealed that conventional laser photocoagulation has some notable side effects, such as permanent retinal scars, macular edema due to excessive burns, choroidal detachment, peripheral visual field loss, delayed dark adaptation, and atrophic creep (33). Most modern laser treatments have focused on innovations that vary laser pulse duration, wavelength, and spot size to address these issues. For example, the semi-automated pattern-scanning retinal photocoagulation system (PASCAL^®^, PAttern SCAn Laser) can quickly apply numerous spots in a defined pattern, thereby improving the accuracy of the treatment using a scanning laser with short pulse duration (34). Thus, PASCAL can reduce treatment time and increase patient comfort while reducing tissue damage to the inner retina (35). Clinical trials have demonstrated that the laser beam is safe and effective, as its wavelength and spatial and temporal modulation can be continuously optimized (36). The pattern-scanning laser can reduce lateral branch tissue damage and fade similar retinal lesions, unlike conventional laser treatment. Furthermore, it considerably reduced the treatment time and pain endured by the patient (37). In clinical practice, a pattern-scanning laser is commonly used to the treat diabetic macular edema.

The pulse duration of selective retinal therapy is shorter than the diffusion time of generated heat. Therefore, high temperature is mainly confined to melanosomes within the retinal pigment epithelium (RPE) cells, thus allowing the selective treatment of RPE cells without the involvement of other cells (38). Multiple clinical trials have validated the safety and efficacy of selective retinal therapy. The subthreshold diode micropulse laser is a new nondestructive form of thermal laser that utilizes a near-infrared diode laser and a sub-microsecond pulse train to provide selective therapeutic targeting of RPE while preserving the neurosensory retina (39). Subthreshold diode micropulse may alter the metabolic activity of the RPE, leading to the release of VEGF and modulation of angiogenesis and vascular leakage without causing any associated retinal damage (40).

In recent years, several clinical laser and delivery platforms have emerged in response to rapid technological advances. For example, endpoint management algorithms have been designed to precisely control laser energy relative to titration levels, which can titrate laser power to the power required to produce nearly invisible retinal burns (41). Image-guided laser treatments, such as “navigational lasers,” use fundus imaging and treatment devices for specific, targeted retinal laser photocoagulation in a predetermined and highly precise manner and are known to play a role in managing diabetic macular edema (42). Light-mediated ultrasound therapy has also been described and used to treat clinically relevant animal models of retinal neovascularization (43). In another clinical trial, researchers successfully developed a noninvasive intelligent wireless far-red/near-infrared luminescent contact lens that reduced retinal vascular hyperpermeability to a statistically significant level by wearing 120 µW light for 15 min three times a week for 8 weeks in a rabbit DR model (44).

However, laser photocoagulation is not effective for all patients, and if not treated properly, it can cause different degrees of adverse reactions such as visual acuity and visual field damage. Although laser therapy can rapidly treat areas of DR lesions, the laser works by increasing vascular permeability, inducing more inflammation and increasing the severity of DME. This adverse effect can be clinically mitigated by pre-laser vitreous cavity injection of anti-VEGF drugs. Conventional laser pulse duration is long (100-200ms), and there is adjacent tissue damage after treatment, causing increased retinal scarring and subretinal fibrosis, leading to some degree of visual field damage and decreased night vision (45). Conventional laser induced transient upregulation of VEGF expression in the retinal neuroepithelium, retinal pigment epithelium and choroid of mice, leading to choroidal neovascularization and DME formation (46). Total retinal photocoagulation does not control optic nerve damage alone, so it is necessary to treat patients with a combination of clinical medications to control their condition and symptoms and further provide protection for their vision. On the whole, laser therapy can reduce the required injections and provide highly effective and long-lasting therapeutic benefits, overcoming many real-life limitations (47). Therefore, research on the mechanisms of laser therapy and the development of new laser treatments with fewer side effects are necessary.

### 3.2 Surgical treatment

Vitrectomy is performed in patients with advanced DR presenting with intravitreal hemorrhage and retinal detachment of retractive or porous origin to prevent vision loss and visual field defects. This surgical technique aims to clear away vitreous body, remove fibrovascular membrane, relieve retinal traction, and inhibit the occurrence and development of vitreoretinal proliferation. Owing to advances in minimally invasive techniques, the original conventional 20G vitrectomy has been upgraded to 23/25/27G+3D minimally invasive vitrectomy that is characterized by smaller surgical incisions and more efficient cutting (48, 49). The use of small-incision vitrectomy and wide-angle viewing systems (e.g., Resight^®^) has led to less invasive and safer vitreoretinal surgery (50). For complex PDR, preoperative intravitreal pretreatment with anti-vascular endothelial growth factor drugs has been shown to reduce the probability of intraoperative and postoperative bleeding, reduce the use rate of electrocoagulation, shorten the operation time, and reduce postoperative complications (51). Vitrectomy is gradually becoming minimally invasive, but there are still some patients with poor visual recovery after surgery, and the process of stripping the proliferating membrane will bleed and may even cause retinal damage. According to clinical data, small-incision vitrectomy can reduce the occurrence of adverse reactions, but some adverse reactions still occur after surgery, such as: paracentral acute middle macular degeneration, postoperative intraocular inflammation, secondary neovascular glaucoma, and postoperative vitreous rebleeding. Vitrectomy combined with intravitreal injection of anti-VEGF drugs or steroids may improve perioperative conditions and reduce the risk of postoperative retinal hyperplasia and complications (52–54).

### 3.3 Drug treatment

#### 3.3.1 anti-VEGF drugs

Vascular endothelial growth factor also plays a key role in angiogenesis and vascular permeability (55). The VEGF protein family includes VEGF-A, VEGF-B, VEGF-C, VEGF-D, and VEGF-E, and the placental growth factor (56). VEGF-A is a crucial regulator of ocular angiogenesis and vascular permeability. VEGF-A mediates angiogenesis by promoting endothelial cell migration, proliferation, and survival and has inflammatory properties through its ability to regulate microvascular permeability and increase leukocyte adhesion (57). Studies have shown that anti-VEGF drugs reduce retinal neovascularization mainly by inhibiting VEGF.

Bevacizumab is a full-length recombinant humanized monoclonal antibody with a high affinity for all VEGF-A isoforms, and its safety and efficacy have been demonstrated in several clinical trials (58). This antibody reduced neovascularization and effectively alleviated vitreous hemorrhage after vitrectomy in patients with PDR (59). Ranibizumab is an affinity-enhancing antibody fragment isolated from bevacizumab with specificity for all subtypes of VEGF-A (58). It reduced the severity of DR and halted its progression, and patients were less likely to experience visual impairment diabetic macular edema and visual field loss compared with PRP (60, 61). Aflibercept is a recombinant fusion protein with a higher affinity for binding to all VEGF-A isoforms than bevacizumab and ranibizumab. It is superior to other anti-VEGF agents in improving baseline visual acuity in patients with PDR (58) (**Table 1**).

**Table 1:** Basic properties and functions of main anti-VEGF drugs.

Anti-VEGF drug	Molecular weight	Molecular structure	Targets	Functions and advantages	References
Bevacizumab	148kDa	VEGF-A Ig-G	VEGF-A	a. Blocks the neovascular stimulus and VEGF-induced increased vascular permeability b. Interacts with HIF-1 reducing VEGF production	Tamura et al. (62)
Ranibizumab	48kDa	VEGF-A Fab	VEGF-A	a. Binds to VEGF-A to form a unique dimer complex with higher stability b. Has great molecular affinity to VEGF	Platania et al. (63)
Aflibercept	115kDa	VEGFR-1 D2+VEGFR-2 D3+Fc	VEGF-A、VEGF-B、PGF	a. Has great binding affinity to VEGF and a long intravitreal half-life relative b. Can antagonize growth factors other than VEGF	Papadopoulos et al. (58)
Faricimab	150kDa	VEGF-A Fab+Ang-2 Fab+Fc	VEGF-A、Ang-2	a. Binds VEGF-A and Ang-2 simultaneously and independently, interfering with the VEGF and Ang-1/Tie2 pathway, thereby preventing the formation and exudation of new blood vessels	Nicolò et al. (64)
Brolucizumab	26kDa	scFv	VEGF-A	a. Has high concentration in a single injection b. Penetrates the retina and choroid and inhibits VEGF-A effectively c. Has low systemic circulation exposure	Tadayoni et al. (65)
Conbercept	143kDa	VEGFR-1 D2+VEGFR-2 D3/D4+Fc	VEGF-A、VEGF-B、VEGF-C、PGF	a. Has high efficiency and stability because of the VEGFR-2 part	Iglicki et al. (66)

In these pivotal trials, most of the functional and anatomic outcomes favored the 0.5 mg dose of ranibizumab compared with the 0.3 mg dose (67). The phase III, double-masked, multicenter, randomized, Active treatment-controlled study of the efficacy and safety of 0.5 mg and 2.0 mg Ranibizumab administered monthly or on an as-needed Basis (PRN) in patients with subfoveal neovascular age-related macular degeneration (HARBOR) study confirmed that ranibizumab 0.5 mg dosed monthly provides optimum results in patients with wet AMD (68). There was no statistically significant difference in the mean number of letters gained between the 0.5 and 2.0 mg RBZ groups through month 6 and 24. In this diabetic macular edema study population, high-dose RBZ does not appear to provide additional benefit over 0.5 mg RBZ (69, 70). To slow disease progression and to maintain visual acuity, in most cases repeated intravitreal injections have to be applied and frequent visits in ophthalmological centers are necessary (71). Generally, to some extent, increasing the dose can improve the efficacy, prolong the action time, and increase the injection interval. However, after reaching a certain degree, increasing the dose may not benefit much and may lead to safety problems. Therefore, it is important to explore the optimal dose of drug.

However, anti-VEGF drugs have a short half-life and require frequent intravitreal injections to ensure efficacy, and interruption of injections may lead to further deterioration of retinopathy in patients (72). To address these problems, novel anti-VEGF drugs, such as brolucizumab, which has a smaller molecular weight, higher unit concentration, longer half-life, and better therapeutic efficacy, have been investigated (73). In addition, AKB-9778 reduced vascular permeability and DR incidence by blocking the activation of Tie-2 via the vascular endothelial protein tyrosine phosphatase, thus activating the Tie-2 tyrosine kinase receptor and leading to the enhancement of connexins and stabilization of the vascular system to limit permeability (74, 75) (**Table 1**).

Traditional treatments of DR often fail to halt disease progression, necessitating multiple intraocular injections. Retreatment depends on disease activity defined based on changes of best corrected visual acuity and lesions detectable in optical coherence tomography (OCT) images. Patients’ compliance and adherence to therapy is crucial for success of treatment, but the unpredictable frequency of visits and administration of IVIs impose a considerable burden on patients. An IVI can be stressful and can generate apprehension of pain and anxiety (71).

Anti-VEGF drugs affect the renal status of patients with DR, leading to new kidney damage or progression of pre-existing kidney disease. Intravitreal injection of anti-VEGF drugs increases the risk of dialysis, by up to 85% compared with untreated patients (76). In the injection of anti-VEGF drugs in patients with DR, it is important to closely monitor the patients’ renal function, urinary protein and creatinine, especially in patients who have multiple injections of the drug in a short period of time. Some PDR patients with intravitreal anti-VEGF therapy progress to detached retina, which is usually characterized by a sudden loss of vision in the affected eye 1 to 6 weeks after intravitreal anti-VEGF injection. However, it has been reported that three patients with PDR had their original detachment of the pulling retina reduced and retinal reset occurred after anti-VEGF treatment (77).

To date, intravitreal anti-angiogenic agents used in clinical practice are based on limited activity against factors belonging to the VEGF family, including ranibizumab, bevacizumab, brolucizumab, as well as aflibercept (78). Angiopoietin-2 (Ang2) plays a critical role in physiologic and pathologic angiogenesis (79). Ang2 is involved in pericyte recruitment, and modulates intraretinal, and preretinal vessel formation in the eye under physiological and pathological conditions (80). Ang-2 and VEGF-A synergistically drive vascular leakage, neovascularization and inflammation, key components of retinal vascular diseases (81). Faricimab, as the first bispecific monoclonal antibody, which simultaneous inhibits of Ang-2 and VEGF-A (82). It demonstrated statistically superior visual acuity gains versus ranibizumab at week 24 in treatment-naïve patients (83). In addition, Faricimab , as revealed in two phase 3 trials over one year, demonstrated robust vision gains and anatomical improvements. The adjustable dosing extending up to every 16 weeks, demonstrating the potential for faricimab to extend the durability of treatment for patients with diabetic macular oedema compared with aflibercept (84, 85). Faricimab T&E exhibited superior retinal drying and fewer injections compared to other treatments with flexible dosing regimens, demonstrating better visual acuity outcomes compared to ranibizumab and bevacizumab (86). Due to its dual mode of action, faricimab may offer therapeutic benefits for patients unresponsive to existing intravitreal therapy. Treatment with faricimab every 8 weeks also demonstrated benefits across various end points, supporting the proportion of participants gaining vision, reduction in visual acuity loss, and anatomical improvements in CNV lesions and CST, supporting the potential of simultaneous Ang-2 and VEGF-A neutralization to reduce treatment frequency.

In addition, treatment with anti-VEGF in patients with PDR with notable fibrosis may increase the risk of developing detached retinal detachment, possibly owing to elevated levels of pro-fibrotic unconjugated connective tissue growth factor (CTGF) resulting from VEGF downregulation. Vascular endothelial growth factor upregulates CTGF. Furthermore, the effects of CTGF were inhibited by the formation of VEGF-CTGF complexes (84). When CTGF levels reach a threshold and trigger a fibrotic response, fibrotic conversion occurs (87). In the future, mutual inhibition of CTGF and VEGF may be a new direction for research and treatment (88)

#### 3.3.2 Steroid drugs

Glucocorticoids have a strong anti-inflammatory effect, which can not only affect the leakage and production of blood vessels, but also block the production of inflammatory cytokines and inhibit VEGF-A-induced leukocyte adhesion. Glucocorticoids can also directly down-regulate VEGF-A expression, increase tight junction protein production and reduce its phosphorylation. In addition it has been shown to be neuroprotective. However, glucocorticoids are extremely anti-inflammatory, but systemic administration has the physiologic effect of increasing blood glucose. Topical glucocorticosteroids are also prone to cataract formation and glucocorticoid-induced glaucoma, so their use in clinical practice is severely limited. (89).

Triamcinolone acetonide (TA) is a water-soluble glucocorticoid antibacterial drug, which is characterized by high efficiency and long-acting properties, and binding to receptors can inhibit the release of inflammatory mediators, slow down neovascularization, and reduce vascular permeability, while it can improve lysosomal membrane stability, block cells from participating in inflammatory responses, and repair inflammatory tissue damage, and is currently the most effective steroid drug for the treatment of PDR. Clinical trials have shown that intravitreal injection of TA after vitrectomy for PDR can improve postoperative visual outcomes (90, 91). A clinical trial showed that TA combined with aminoguanidine inhibits inflammation and oxidative stress, improves vascular endothelial function and retinal function, and reduces VEGF expression in patients with DR (92). In addition, a study found that dendrimer-conjugated TA was more effective than free TA in terms of anti-inflammatory effects and had the key benefit of reducing side effects (93). Further, dendrimer-conjugated TA inhibited inflammatory cytokine production, microglia activation, and pre-retinal neovascularization in oxygen-induced retinopathy (OIR) and improved neuroretinal and visual dysfunction caused by OIR (94).

#### 3.3.3 Senolytics drugs

During DR progression, pericytes and endothelial cells undergo continuous apoptosis owing to the accumulation of various inflammatory and growth factors. The pathological vascular system of the DR retina can work together with the cell cycle inhibitory protein p16 INK4A and the anti-apoptotic protein BCL-xL to limit the retina’s ability to clear senescent retinal cells (95). To make matters worse, senescent cells release products that cause inflammation and death of non-senescent cells, known as senescence-associated secretory phenotypes, thus increasing the risk of DR progression (96).

Senolytic drugs eliminates senescent cells (cells that resist apoptosis and do not divide but remain metabolically active) from various tissues in the body without damaging or destroying healthy tissues. Senolytic drugs currently in clinical trials include the drug combination dasatinib and quercetin, Fisetin, and UBX-1325, an inhibitor of the pro-apoptotic protein BCL-xL, specifically designed to combat age-related ocular diseases such as PDR, age-related macular degeneration, and other diseases involving ocular vascular dysfunction (97). UBX-1325 also improved the retinal vascular system in animal models of OIR and streptozotocin-induced diabetes (98). Although senolytic and anti-aging drugs may have severe unknown side effects, they have great potential in combatting age-related diseases (99). In a diabetic mouse model, D + Q reduced the occurrence of diabetic complications. the number of hepatic senescent cells and renal senescent cells was significantly decreased in D + Q mice, and the incidence of cirrhosis and proteinuria was reduced (100, 101). In addition, D+Q ameliorated and reduced neuroinflammation and minimized nerve damage in diabetic mice. Thus, the anti-aging drug not only restores peripheral insulin responsiveness in diabetic mice, but also attenuates diabetic complications for which there is currently no effective mechanism for treatment.

Another way to enhance immune clearance of senescent cells is to enhance the activity and increase the accumulation of immune cells responsible for surveillance of senescent cells. A more subtle stimulation of the immune system using specific cytokines that enhance NK cells may be a viable approach. The cytokines IL-21 and IL-15 are thought to significantly enhance NK cell-mediated antitumor immunity. However, the role of these cytokines on immune surveillance of senescent cells has not been elucidated, and their effectiveness as inhibitors of senescence needs to be tested in disease models (102).

As our research into the characterization of senescent cells *in vitro* and *in vivo* continues to deepen, many challenges remain for anti-aging drugs. For example, there is a very high degree of heterogeneity in the senescence state of different cells upon stimulation, and cellular senescence is a dynamic process that evolves over time, yet we have not yet clarified the cell-loaded “senescence phenotype” *in vivo*. We need universal senescence markers or specific markers to clearly isolate and characterize senescent cells. At the same time, we need to clarify the similarities and differences between cellular differentiation and senescence-regulated processes, as well as the strong paracrine effects of senescent cells on non-senescent cells, so that anti-aging drugs can specifically identify and target specific subpopulations of senescent cells that are the most deleterious to tissue function, and thus minimize the deleterious effects. For the time being, a single-cell transcriptomics approach, including spatial transcriptomics, is the only solution to this dilemma, but it requires a great deal of work.

Most of the current studies on senescent cells are limited to animal models, and it is not possible to systematically assess whether the elimination of senescent cells under prolonged action of anti-aging drugs will have long-term toxic effects or negative consequences. The safety and efficacy of anti-aging drugs in humans are still not fully proven, and their long-term effects on the human immune system require further research. In addition, utilizing the intrinsic surveillance ability of the autoimmune system to target senescent cells may be a promising therapeutic modality. Therefore, treating DR with senolytics may be a promising research direction.

#### 3.3.4 Retinal ganglion cell injury and regeneration-related drugs

Elevated oxidative stress in the retina plays a crucial role in causing RGC injury. ROS and reactive nitrogen species produced during oxidative stress can activate multiple metabolic pathways, including increased fluxes of the polyol and amino sugar pathways, leading to excessive activation of protein kinase C isoforms, accumulation of AGEs, and acceleration of RGC injury (103). Conversely, reducing oxidative stress by inhibiting ROS levels in the retina can considerably improve RGC function. The nuclear factor erythroid 2-related factor 2 not only regulates the inflammatory response by regulating the expression of NF-κβ and cyclooxygenase-2 but also prevents ROS accumulation by inhibiting the activation of NAD(P) oxidase 2. It also plays a crucial role in mitochondrial homeostasis (104). In a model of OIR, nuclear factor erythroid 2-related factor 2 can promote restorative angiogenesis in late diabetic stages by regulating NAD(P) oxidase 2 (105).

Epigallocatechin-3-gallate has strong antioxidant properties and downregulates ROS/thioredoxin Interaction protein/NOD-like receptor protein 3 inflammasome axis activity, alleviates oxidative stress, and inhibits the production of pro-angiogenic factors (106). In addition, it reduces reactive gliosis in Müller cells and retinal damage, with potential neuroprotective effects (107). Curcumin can exert antioxidant effects and alleviate diabetic retinal damage by inhibiting the AGE-RAGE signaling pathway and extracellular matrix receptor interactions in the diabetic retina (108). Resveratrol can activate the AMPK/SIRT1/PGC-1α pathway, scavenge high glucose-induced intracellular ROS, reduce AGE levels, exert antioxidant effects, and reduce retinal inflammation and damage (109). Resveratrol also inhibits diabetes-induced Müller cell apoptosis through the MicroRNA-29b/specific protein 1 pathway (110). Luteolin has powerful antioxidant effects and can effectively inhibit oxidative stress-induced apoptosis of retinal ganglion and pigment epithelial cells (111, 112). Supplementation with various vitamins in the diet can improve oxidative stress in the retina caused by hyperglycemia and inhibit the progression of DR (113).

Aldose reductase (AR) is a rate-limiting enzyme in the polyol metabolic pathway. Sustained high glucose levels lead to AR production and accumulation, followed by activation of the polyol metabolic pathway, resulting in intercellular sorbitol accumulation, generation of AGEs, and hypoxia (114). In addition, AR promotes neovascularization by increasing the expression of inflammatory cytokines and VEGF through the activation of nuclear factor κβ (NF-κβ) (115). This may be involved in the process of RGC damage in DR. Aldose reductase inhibitors (ARIs) can attenuate RGC damage by inhibiting extracellular signal-regulated kinase 1/2 phosphorylation and NF-κβ and VEGF expression (116). Several studies have demonstrated that ARIs exhibit neuroprotective effects in the retina and inhibit retinal capillary basement membrane thickening (117). However, owing to the non-specific binding of ARIs to the members of the aldo-ketoreductase protein family, which have a high structural similarity to AR, ARIs have many adverse side effects. Therefore, developing specific ARIs may be a new and effective way to prevent and treat RGC injury.

Pituitary adenylate cyclase-activating polypeptide is a neuroprotective neuropeptide that protects retinal cells from ischemia-induced oxidative stress and has antioxidant, anti-apoptotic, and retinal-protective effects (118). It is expected to be a new approach to DR treatment. Neurotrophic factors include brain-derived neurotrophic factor, nerve growth factor, NT-3, NT-4, taurodeoxycholic acid, and glial cell line-derived neurotrophic factor and have potential protective effects on the survival of RGCs in animal models of DR (119, 120). However, their clinical application faces potential difficulties, such as the administration of neurotrophic agents, source and extraction techniques, storage, and possible side effects, thus requiring further research.

Recent advancements in exosome and stem cell transplantation techniques show promise in regenerating RGCs after injury. In conclusion, the protective therapy of RGCs is an effective way to prevent the progression of DR. Furthermore, identifying the mechanism of RGCs injury and finding breakthrough targets are essential to achieve the purpose of effectively protecting RGCs.

#### 3.3.5 Other drugs

Fenofibrate is a peroxisome proliferator-activated receptor alpha (PPARα) agonist. Numerous experimental studies have confirmed the beneficial effects of fenofibrate in improving retinal vascular leakage and leukoplakia, downregulating the expression of VEGF, and reducing endothelial and pericyte loss (121). Fenofibrate exhibited positive effects in several experimental animal models of DR (122). Some novel PPARα agonists, such as pemafibrate and 7-chloro-8-methyl-2-phenylquinoline-4-carboxylic acid (Y-0452), have shown satisfactory effects in animal models (123). Therefore, the use of PPARα agonists may be a promising therapeutic approach for preventing the development and progression of DR.

Long-term follow-up studies have shown that intensive glycemic control in patients with diabetes can slow the progression of microvascular complications, including retinopathy, nephropathy, and neuropathy. Several clinical trials have reported that sodium-glucose cotransporter-2 (SGLT2) inhibitors can reduce the incidence of macrovascular and microvascular complications in diabetes through revascularization (124). SGLT2 inhibitors in DR model mice considerably inhibited oxidative stress, apoptosis, and restoration of tight junctions in the retina, downregulated inflammatory and angiogenic factors, and improved DR symptoms (125). Furthermore, peripapillary cells express SGLT2, therefore, SGLT2 inhibitors may directly protect against peripapillary cell swelling and loss and prevent retinal hyperperfusion under high glucose conditions (126). However, the long-term efficacy of SGLT2 inhibitors requires further study.

In addition, angiotensin-converting enzyme inhibitors or angiotensin II type 1 or type 2 receptor antagonists can reduce the expression of VEGF and its receptors in the retina. Drugs for microcirculatory disorders can improve retinal blood flow and correct hypoxia with satisfactory efficacy in NPDR. Vascular endothelial protective drugs can inhibit oxidative stress and inflammation, reduce blood-retinal barrier (BRB) injury, and improve vascular permeability and leakage. Hypoxia-inducible factor-1 reduced the expression of interleukin 6 and tumor necrosis factor α in DR, as well as that of miR-125b-5p and miR-146a-5p in the retina, and upregulated the expression of VEGF (127). Thus, targeting hypoxia-inducible factor-1 antagonists in hypoxic tissues may be an excellent approach to preventing DR.

### 3.4 Gene treatment

In the last few decades, gene therapy has made substantial progress, especially in the field of ophthalmology. Eyes are more easily immunologically privileged by injection than other organs due to the BRB. A growing body of data from clinical studies has demonstrated the efficacy and safety of gene therapy in treating inherited eye diseases (128). These results prompted an increasing number of investigators to explore the use of gene therapy in the treatment of non-genetic eye diseases, such as glaucoma, neovascular age-related macular degeneration, and DR (129).

Anti-angiogenic proteins or non-coding RNA interference effector molecules delivered via viral or non-viral vectors effectively inhibit the progression of DR. A study showed that retinal neovascularization was considerably reduced in an animal model of DR after injection of adeno-associated virus (AAV)-mediated anti-VEGF endoreceptor Flt23k (130). Similarly, sFlt-1, a soluble splice variant of VEGF receptor 1, is a potent potential target (131). The introduction of AAV-mediated endogenous angiogenesis inhibitors, such as pigment epithelium-derived factor, angiogestin, endostatin, and calreticulin anti-angiogenic domain in OIR mouse models, can effectively inhibit neovascularization (132). Neovascularization can also be considerably inhibited by the injection of siRNA expression plasmids targeting VEGF in animal models of DR (133). Gene therapy can also protect the retinal vasculature and neurons from damage in the early stages of DR before the appearance of an obvious clinicopathology. An *in vivo* study using AAV vectors encoding small hairpin RNAs targeting early growth response 1, an artificial RNA molecule that can be used to silence the expression of target genes by RNA interference, showed that retinal transduction of these vectors considerably reduced apoptosis in cells located in the inner and outer nuclear layers (134).

Although gene therapy has made great progress in recent years and provided new means for DR treatment, it still faces many challenges. Viral vectors may produce problems such as immune response, off-target effects, inflammation and insertional mutagenesis, and non-viral vectors are safer than viral vectors, but gene transfection efficiency is relatively low. Moreover, gene therapy has potential genotoxicity and requires further long-term monitoring after treatment to assess the safety of this vector. If a new vector with safer, more effective, strong targeting and less toxicity can be developed, gene therapy will usher in a new round of development opportunities and obtain a broader application space.

## 4 Drug-delivery system

**Figure 2:**
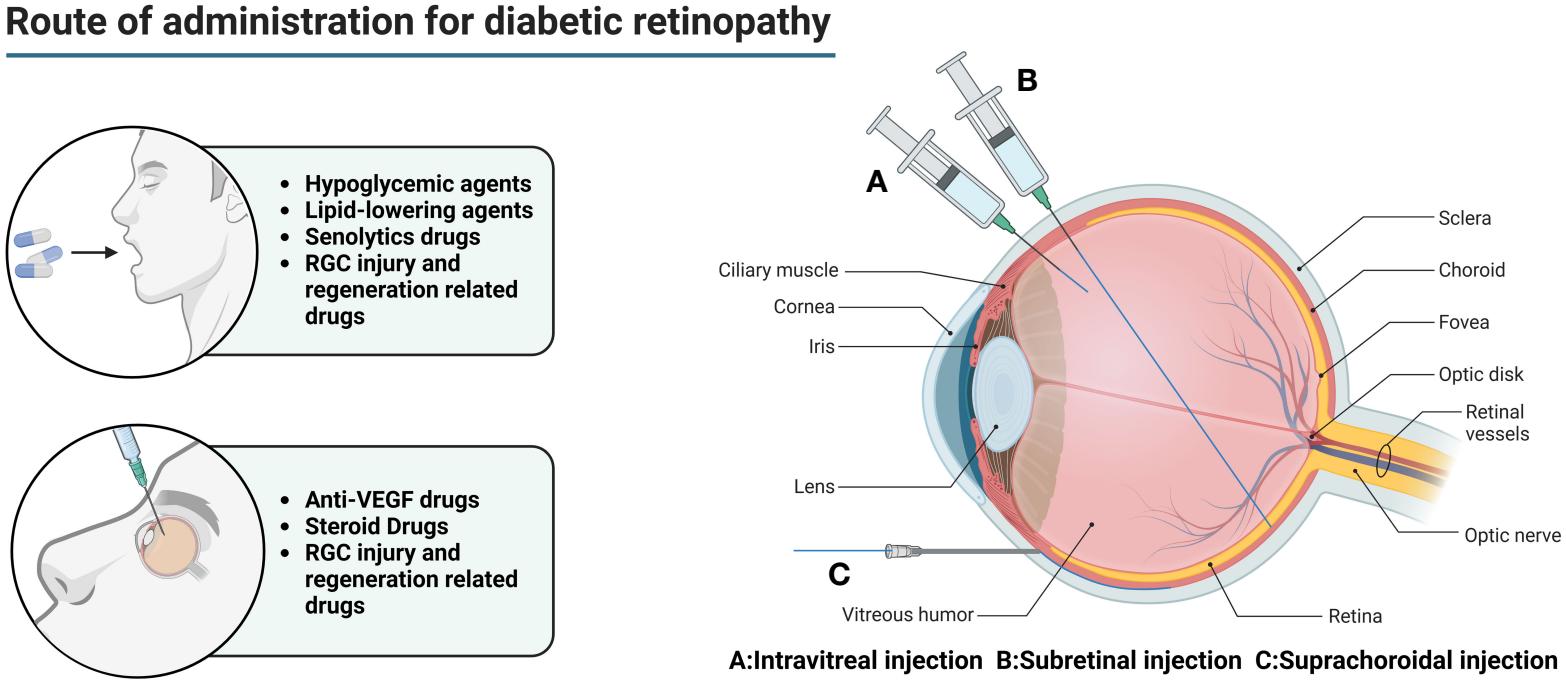
Main routes of administration for diabetic retinopathy. In the early stage of DR, oral hypoglycemic drugs and lipid-lowering drugs are mainly used to reduce the chronic damage of hyperglycemia and hyperlipidemia to the retina. However, this does not completely stop the progress of the disease. For DEM patients, anti-VEGF drugs can be injected into the eye to relieve macular edema and prevent visual impairment. For patients with advanced PDR, combined use of steroid drugs can better improve tissue damage. At the same time, the appropriate amount of senolytics drugs can be used to eliminate senescent cells and their related products. For patients with visual impairment, drugs related to RGC injury and regeneration can be used to promote optic nerve regeneration.

A variety of absorption barriers exist in the eye, mainly including the corneal barrier, conjunctival barrier, blood-atrial barrier (BAB), vitreous, BRB, blood-optic nerve barrier, and tear clearance (135) (**Figure 2**).

Topical drug delivery, such as eye drops, ointment, gel, etc., although can achieve some effect, but due to the influence of the corneal barrier, blood-atrial barrier (BAB), and tear clearance, the bioavailability of the drug is usually <5%, so that topical drug delivery is nearly ineffective for the posterior segment of the eye in diseases such as DR.

Intraocular injections (including intravitreal, subretinal, and suprachoroidal injections) are used to deliver drugs to the posterior segment of the eye in DR patients. While intraocular injections effectively bypass the absorption barrier in the anterior segment of the eye, allowing the drug to reach the eye directly. However, intravitreal injection is a highly invasive method of drug delivery, which not only requires a high level of drug delivery technique, but also is prone to complications such as ocular hemorrhage, pain, increased intraocular pressure, retinal detachment, cataract, endophthalmitis, etc., and frequent injections lead to poor patient compliance (136).

Vitreous cavity injection is relatively safe and simple, but limited by the inner boundary membrane, the drug can diffuse and accumulate in the entire anterior and posterior segment of the eye tissues, and it is difficult to achieve a certain concentration of drug delivery in the outer retina and the RPE, so patients often need multiple injections, which reduces patient compliance and increases the risk of intraocular complications, such as intraocular inflammation, increased intraocular pressure, retinal detachment, and so on.

Subretinal injections are much smaller in dose and the drug can directly contact the photoreceptor cells and the RPE, which is theoretically a safer method of injection due to the enclosed space, immunity, and so on. However, there are often many complications due to the difficulty of the procedure. If the needle is inserted too deeply, it can lead to damage to the RPE, suprachoroidal lumen administration, suprachoroidal lumen hemorrhage, and needle tip obstruction (which is a greater risk of occurring in those with retinal atrophy). If the needle is too shallow, it can lead to intraretinal hydration and retinal belly splitting. Improper manipulation may also result in the formation of subretinal blisters, leading to limited retinal neurosensory damage, extended retinotomy, and postoperative susceptibility to macular tear, persistent retinal detachment, choroidal neovascularization, increased intraocular pressure, cataracts, retinal hemorrhage, endophthalmitis, thinning of the outer nuclear layer, reduced concentration of the drug in the subretinal caused by the reflux of carrier suspension into the vitreous cavity, immune reactions, and decreased visual acuity.

The main modes of drug delivery in the suprachoroidal space include catheter delivery, subcutaneous needle delivery, surgical implant delivery, and microinjector delivery. Catheterized drug delivery is operated under a microscope, which allows for more accurate localization, but it is more difficult to operate and is prone to damage to ocular tissues. Hypodermic needle administration is less invasive and does not require a scleral incision, but requires a high degree of surgical precision and carries the risk of inadvertent injection into the vitreous humor, subretinal space, or subconjunctival space. Surgical implantation requires a large scleral incision, which is invasive and carries a higher risk of repeated administration. Microsyringe administration is simple and less invasive, but there are inter-individual differences between patients, with varying scleral thicknesses that are not easily adjusted.

Periocular administration is less invasive than IVT, but the diffusion of the drug in the sclera is passive, and the amount of drug absorbed into the posterior segment is limited by the nature of the sclera and the nature of the drug.

Systemic administration of the drug is affected by the blood-retinal barrier, making it difficult for the drug to penetrate into the eye, and the bioavailability is extremely low, with only 1% to 2% of the drug reaching the vitreous. Large doses must be given to ensure that the drug entering the eye reaches the therapeutic concentration, and there is a potential risk of systemic adverse effects.

In recent years, researchers have developed nanocarrier drug delivery platforms designed to enhance their permeability, extend their retention time at target sites, and provide sustained release with low toxicity and high efficacy (9). Drug-loaded nanoparticles have many advantages over traditional delivery systems, leveraging nanotechnology’s benefits, including small diameter and good biocompatibility. Nanomaterials can also cross the retina more easily by modulating the physicochemical properties, structure, or affinity groups on the surface of the nanomaterials BRB. Including the drug inside or attached to the nanoparticle surface, the use of nanoparticles can bind to specific receptors on the RPE, so that the drug can pass through the BRB in a targeted manner and reach the epithelial cells, thus achieving the goal of DR treatment. In addition, a nanomaterial can contain multiple drugs, which can deliver two or more drugs to the retina in an optimal ratio, thus obtaining a better combined therapeutic effect. At the same time, the biochemical properties and degradation time of nanoparticles are used to control drug release and provide targeted delivery to specific cells or tissues, thereby prolonging drug half-life, reducing adverse effects, and increasing the route of water-soluble and macromolecular drugs into tissues (137). Nanotechnology can also integrate the physical characteristics of nanomaterials into therapeutic approaches, which is not possible with small molecule drugs. For example, using graphene’s electrical conductivity to treat optic nerve injury models reduces the death of RGCs and directs the growth of RGCs’ axons in the direction of the nanofibers, which opens up a new pathway for the growth of RGCs and the regeneration of optic nerves (138).

### 4.1 Nanoliposomes

Nanoliposomes are small vesicles composed of single or multiple lipid bilayers with an aqueous core in the center and are used as drug-delivery vehicles for both lipophilic (loaded in the bilayer) and hydrophilic (loaded in the core) drugs (139) (**Table 2**). Compared to drugs alone, drug-loaded nanoliposomes are smaller and more biocompatible, which helps the drug reach the target site with a longer residence time, drug half-life, and contact time with the target site (145). Numerous studies have shown that they function well in the eye (146). In a study, intravenous injection of cyclosporine A-loaded lipid nanocapsules into DR mice broke the inflammatory cycle, reduced microglial activation and macrophage recruitment, and inhibited pro-inflammatory cytokine release (147). Nevertheless, despite their great potential as drug-delivery carriers targeting the retina, the use of nanoliposomes has revealed some drawbacks, such as blurred vision, induction of inflammation during cationic liposome delivery, and the tendency to aggregate *in vivo* owing to poor colloidal stability.

**Table 2:** Advantages and structural schematics of the main nano-delivery carriers.

Nano-delivery carriers		Advantages	Structure	References
Nanoliposomes		● Small volume ● Excellent biocompatibility ● Longer residence time ● Longer drug half-life	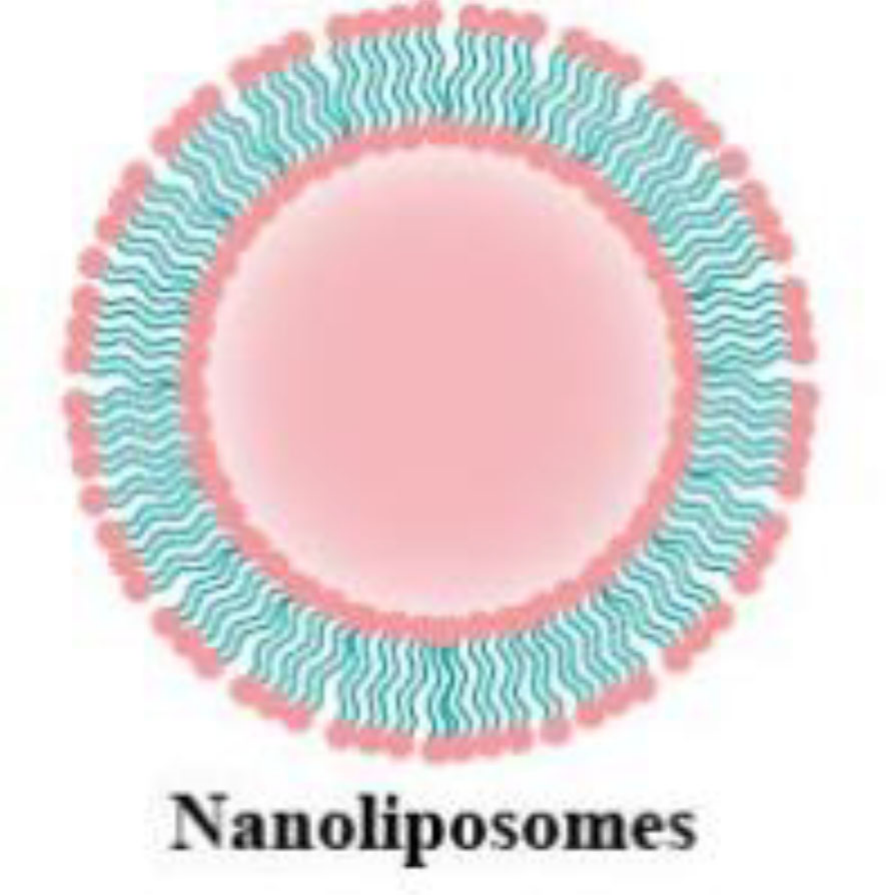	Akbarzadeh et al. (139)
Nanoparticles	Polymeric Nanoparticles	● Biocompatibility ● Biodegradable ● Non-toxic degradation products	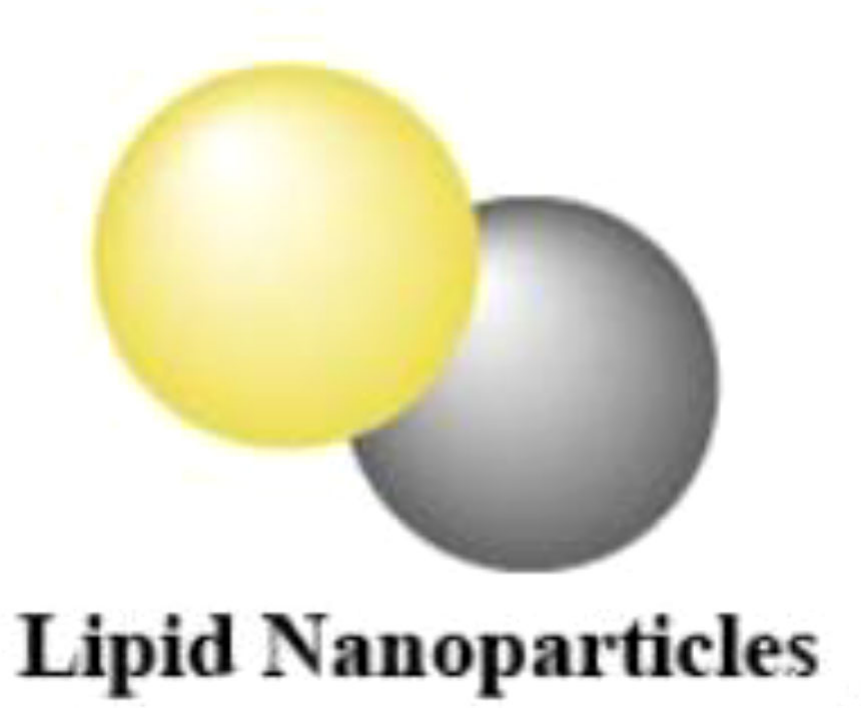	Tahara et al. (140)
	Lipid Nanoparticles	● Small volume ● High stability ● Excellent biocompatibility ● Crosses biological barriers ● High and sustained drug release ● Targeted drug delivery		Sharma et al. (141)
Nanoemulsions	o/w Nanoemulsions	● Small volume ● Excellent biocompatibility ● Crosses biological barriers ● Targeted drug delivery	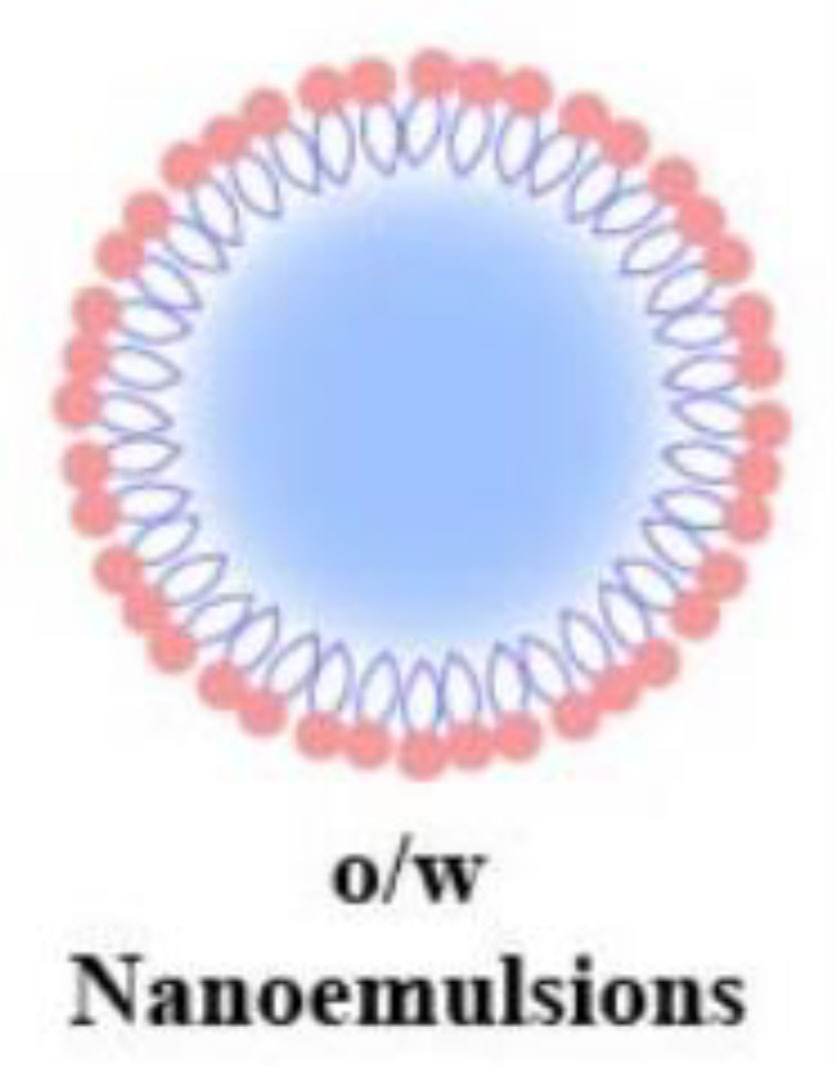	Singh et al. (142)
	w/o Nanoemulsions		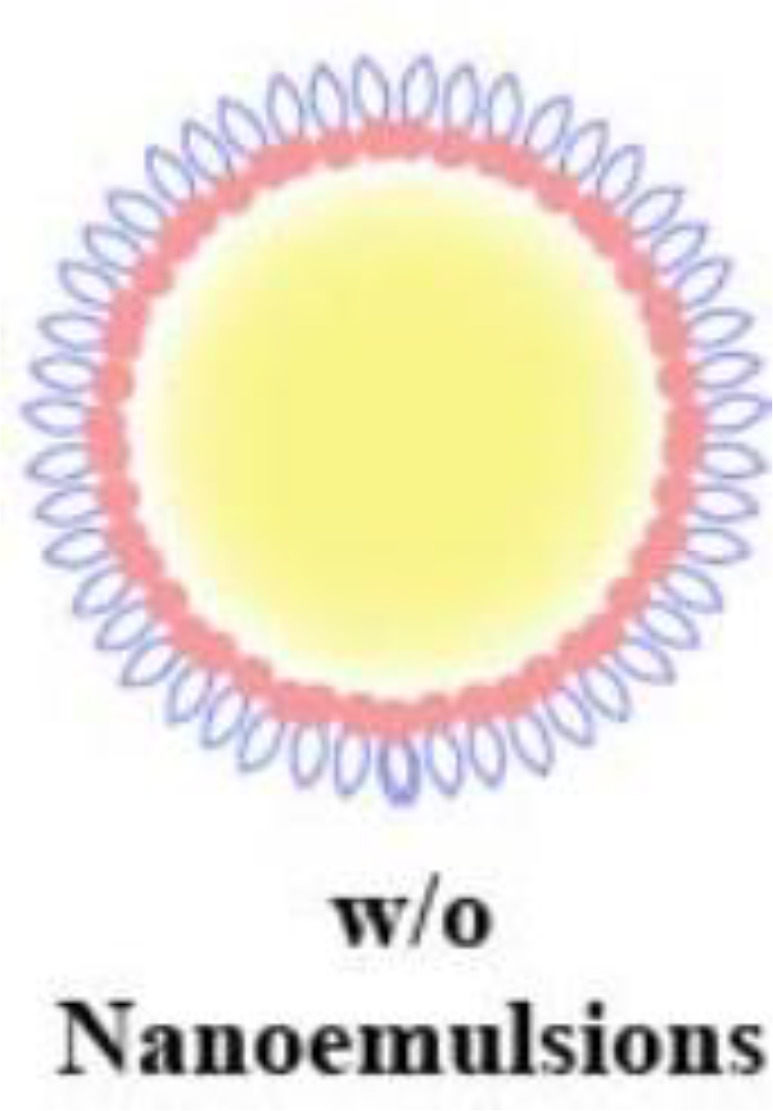	
Nanogels	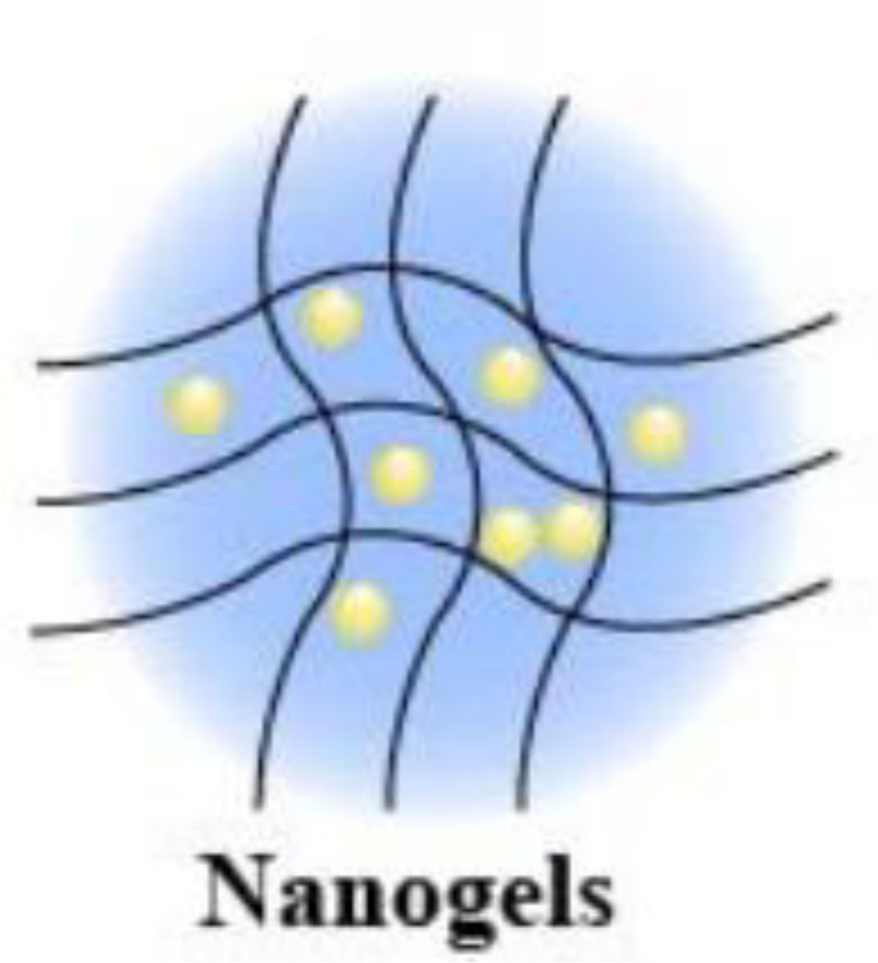	Dave et al. (143)
Dendrimers		● High stability ● Improving ocular permeability ● High bioavailability of drugs	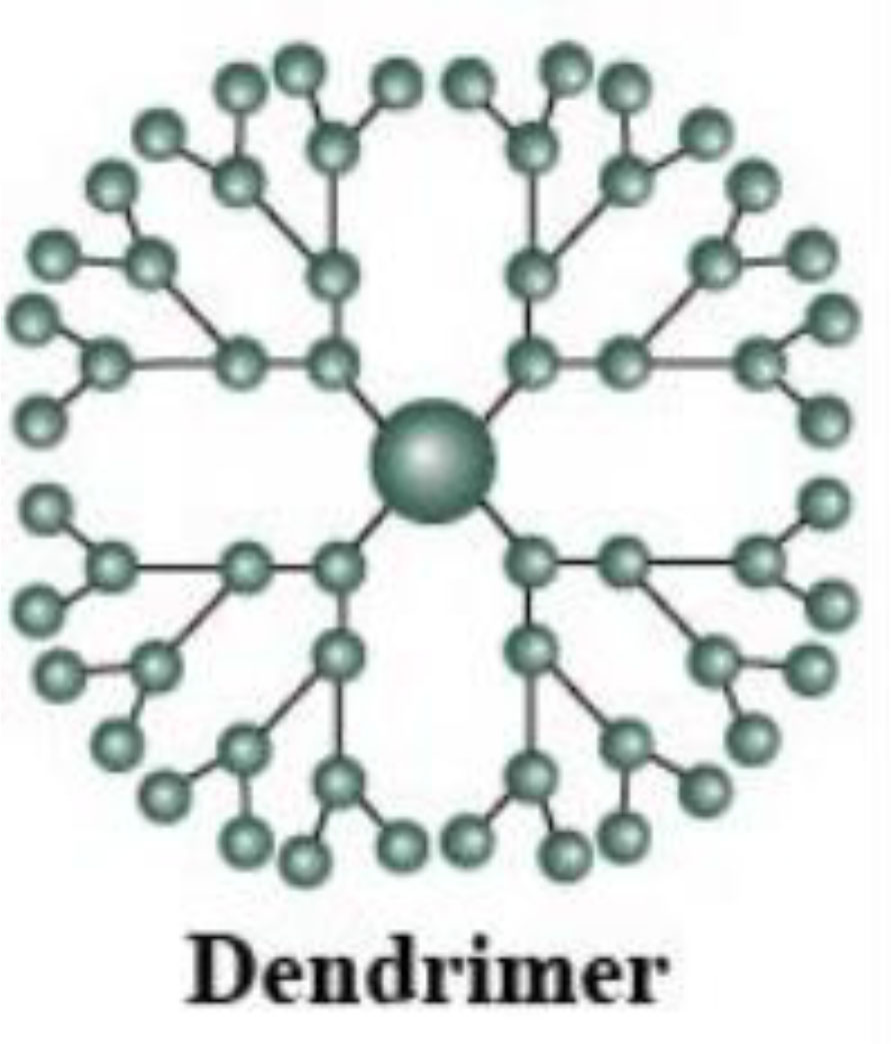	Yavuz et al. (144)

### 4.2 Nanoparticles

Nanoparticles (NPs) are mainly classified as polymeric or lipid NPs (**Table 2**). They are approved by the US FDA for clinical use owing to the biocompatibility, biodegradability, and non-toxicity of the degradation products of poly-lactic-co-glycolic acid (140). Surface-modified poly-lactic-co-glycolic acid-NPs loaded with pioglitazone can deliver drugs to the posterior segment of the eye after intraocular topical administration in diabetic rats, and VEGF levels in suspension were notably reduced (148). A novel insulin delivery system, chitosan nanoparticles/poly-lactide-co-hydroxyacetic acid-poly-ethylene glycol-poly-lactide-co-hydroxyacetic acid hydrogel was developed. This system exhibited adequate neuroprotective effects on the retina of DR rats via subconjunctival injection, facilitating controlled insulin delivery (149). Human serum albumin is a major endogenous soluble protein that serves as a reservoir for the transport of many different compounds, and showed increased solubility, reduced toxicity, antioxidant protection, and prolonged half-life *in vivo*. Human serum albumin-nanoparticles combined with polyethylene glycol loaded with apatinib effectively inhibited VEGF-induced retinal vascular hyperpermeability both *in vitro* and *in vivo* (150). Hyaluronic acid (HA) is a degradable, non-immunogenic biopolymer that is a major component of vitreous humor and interacts with many retinal cell surface receptors. Degradation of endothelial HA was observed during DR pathophysiology. A study developed an HA-encapsulated bovine serum albumin NP-based delivery system for the topical administration of apatinib, a novel selective VEGF receptor 2 inhibitor. The system could be used as a platform for enhanced topical treatment of DR to overcome disruptive intravitreal pathway ocular complications (151).

Lipid NPs mainly include solid lipid nanoparticles (SLNs) and nanostructured lipid carriers (NLCs). SLNs comprise solid lipids, surfactants, and co-surfactants and can be loaded with hydrophilic or lipophilic drugs. NLCs are typically prepared from solid- or liquid-type lipids. SLNs and NLCs have the advantages of small size, high stability, excellent biocompatibility, ability to cross biological barriers, high and sustained drug release, and targeted drug delivery. In a rat model of DR, a modified melt emulsification-sonication method for preparing chitosan-modified 5-fluorouracil NLCs provided higher and sustained drug release and superior anti-angiogenic effects (141).

Additionally, the growth inhibitor analog octreotide (OCT) was combined with magnetic NPs, and their potential as an OCT delivery system for treating retinal pathologies, such as DR, was investigated. The magnetic NP-OCT can act as an intraocular delivery system for OCT, ensuring OCT localization to the retina and enhancing the biological activity of OCT (152).

### 4.3 Other nano-delivery technologies

Nanoemulsions are drug-loaded in oil-in-water or water-in-oil emulsions that are stable with different surface activities. The drug penetrates the ocular tissue with the emulsion and is released at the target site (**Table 2**). Nanogels are nano-sized hydrogels comprising hydrophilic polymers that can cross the ocular barrier, thus delivering the drug to the posterior part of the eye. Dendrimers are star-shaped, highly branched, and water-soluble macromolecular systems, in which drugs can bind to dendrimers through hydrogen bonding, hydrophobic interactions, covalent bonds, and ionic interactions (143). Dendrimers using poly-amidoamine as a structural basis are the most commonly used for ocular drug delivery, and subconjunctival injection of dexamethasone-poly-amidoamine conjugates improved ocular permeability and bioavailability of dexamethasone in ocular tissues (144). Quercetin is a bioactive plant flavonol compound with potential free radical scavenging and neuroprotective effects, but it is limited by poor bioavailability and a variety of biological interactions. nano-formulation of quercetin has been shown to have hypoglycemic effects, modulate homocysteine pathways, reduce lipid peroxidation, and scavenge free radicals in zebrafish DR models (153).

It was found that sSema4D/PlexinB1 signaling can regulate pathological neovascularization and leakage in DR. Sema4D/PlexinB1 -mDIA1 signaling affects endothelial cell migration, proliferation, and regulates endothelial cell phosphorylation and pericyte internalization leading to vascular leakage. Nanopeptide eye drops developed for Sema4D were shown to efficiently penetrate the ocular surface into the retina and vitreous humor through positive electrostatic attraction and small particle size penetration, and specifically recognize and precisely target sSema 4D in the vitreous humor and retinal region. smart peptides subsequently self-assembled to form a fibrous network wrapped around sSema4D, achieving smart capture of sSema4D and blocking the interaction of sSema4D with three receptors. 4D binding to the three receptors, achieving a significant reduction in pathologic retinal neovascularization and leakage. What’s more, this study confirmed that in DR treatment, non-invasive SSP eye drops can achieve the same therapeutic effect as invasive antibody injections, when SSP eye drops combined with anti-VEGF can increase the current clinical therapeutic effect by 50% (154).

Curcumin (CUR) and many other plant-derived polyphenolic compounds are known to act on multiple signaling pathways simultaneously; however, most of them have low bioavailability after oral administration, resulting in poor clinical efficacy. Based on this development, orally bioavailable nanocurcumin, in combination with insulin glargine, inhibits the production of multiple inflammatory factors and prevents or delays the progression of DR in a diabetic rat model (155).

In another study, the authors’ team constructed a system using cyanobacteria as a carrier loaded with gold nanoparticles (Au NPs) with glucose oxidase-like activity and iridium nanoparticles (Ir NPs) with catalase-like activity (Cyano@Au@Ir). In this system, Au NPs nano-enzymes first degrade glucose into hydrogen peroxide, which is then decomposed into H2O and O2 by Ir NPs, completing the cascade glucose-lowering reaction. Due to the light transmittance of the eye and light accumulation in the retinal region, cyanobacteria can continuously produce oxygen to alleviate the hypoxic state of the microenvironment, which contributes to the reduction of the expression of vascular growth factor and hypoxia-inducible factor. Meanwhile, Ir NPs can eliminate the highly expressed peroxides in the DR microenvironment and play an anti-inflammatory role. In a DR animal model, Cyano@Au@Ir significantly reduced neovascularization and vascular leakage. This novel therapeutic modality provides a continuous supply of oxygen, scavenges free radicals, lowers blood glucose, and performs comprehensive microenvironmental regulation, which provides a new perspective for solving DR fundus complications.

Despite the tremendous progress that has been made in nanotechnology, numerous scientific challenges remain. Nanoparticles are composite structures, and their composition, assembly mode, particle surface, ligands, rigidity and charge properties all affect the properties of the structure. The complexity of nanoparticles makes it difficult to analyze their potential toxicity, and there are no suitable models for the evaluation and study of nanobiological interactions (especially nano-immune interactions). The determination of optimal physicochemical parameters is crucial for the successful development of therapeutic nanoparticles. However, ensuring reproducibility and transparency severely limits the systematic and large-scale screening of nanoparticles due to the difficulty of synthesizing nanoparticles with different properties in a rapid, precise and reproducible manner.

The most important challenges for the clinical translation of nanomedicines still derive from a lack of understanding of biological complexity and individual heterogeneity, limited understanding of nanostructure-biological interface interactions, and lack of reproducible synthesis and large-scale production technologies for nanomedicines. While nanomedicines in the future are developing towards finer design, more in-depth studies on the mechanism of nanomedicines and cellular interactions are needed to regulate cellular uptake of drugs by regulating related target molecules, and in addition, nanomaterials with good biosafety and clear metabolic behavior *in vivo* should be selected as much as possible. Even though nanoparticles are promising, labor and material costs are more expensive, and it is still difficult to standardize the quality standards of the products. In order to improve drug delivery of nanomaterials, we need to better understand not only the *in vivo* processes of nanomaterials in different routes of drug delivery, but also the relationship between the physicochemical properties of nanoparticles and the desired biological effects.

## 5 Conclusion and future perspectives

In recent decades, researchers have been working to elucidate the pathogenesis of DR and have been hoping to identify breakthrough drug targets to effectively inhibit its onset and progression. Improvements and innovations in laser technology and surgical approaches have also emerged. This review briefly introduces the pathogenesis of DR, summarizes the current treatments for DR, and provides an overview of the latest advances in various methods, including laser, surgical, and drug treatments. Generally, patients with severe NPDR and mild-to-moderate PDR can be treated with laser therapy or anti-VEGF therapy, whereas those with severe PDR require surgical treatment to remove the vitreous. However, no matter at any stage, effective control of blood glucose, blood pressure and blood lipids is crucial to prevent the occurrence and development of DR. Therefore, effective control, treatment and intervention of relevant metabolic abnormalities in diabetic patients are the basis and key steps to ensure the therapeutic effect of DR.

In addition, DR is no longer just a simple microangiopathy but a highly specific neurovascular lesion. At present, the main drugs used in clinical treatment are drugs to improve microcirculation and VEGF inhibitors, but the overall efficacy is not ideal and cannot effectively prevent disease progression. Therefore, there is an urgent need to develop drugs for DR neuroprotection and prevention of injury or promotion of RGCs regeneration to prevent the occurrence of severe visual loss and blindness in the late stage of DR. Protecting the retinal neuro-vascular unit, anti-inflammation and anti-oxidation are all important links in the treatment of DR. Combining drugs targeting different aspects holds promise for benefitting more DR patients.

Gene therapy offers many advantages over long-term injectable anti-VEGF drugs and serves as a promising, attractive, and cost-effective alternative treatment. Therefore, further studies on the pathophysiology of DR are essential to expand the application of gene therapy. Future studies should confirm the effectiveness and genotoxicity of the long-term expression of transgenic, viral, or non-viral delivery vectors. Determination of the optimal intervention period is also vital to achieve the best therapeutic outcome using gene therapy, as irreversible lesions may occur later (156).

Our review describes the current limitations of DR drug delivery and latest advances in nanotechnology for ocular drug delivery. The emergence and continuous innovation of nanotechnology-based drug-delivery systems have led to tremendous improvements in treating DR patients over the past few decades. These nanocarriers can significantly improve the solubility and biocompatibility of drugs, prolong drug residence time, assist a variety of drug components non-invasively, and target specific links in the progression of DR, and nanomaterial-based drug delivery systems have great potential for the application in the treatment of DR.

Nanotechnology also shows promise in DR prevention, early diagnosis, and monitoring of treatment outcomes. In a recent study, researchers have developed a high-brightness adhesive fluorescent nanoprobe targeting VEGFR-2 using biodegradable materials. Following systemic injection, nanoprobes can cross the blood-retinal barrier, bind to microvascular endothelium, and firmly adhere to leukocytes, demonstrating their potential in the early diagnosis of DR. However, the long-term stability, safety, and the possibility of unknown adverse effects of nanocarriers remain a considerable challenge.

In recent years, there has been an increasing emphasis on individualized treatment of patients with diabetes. Digital innovations, including the further integration of telehealth, development of fifth-generation wireless networks, artificial intelligence approaches such as machine learning and deep learning, the Internet of Things, and digital security capabilities, have created new opportunities for individualized patient care. In the future, physicians need to adapt to technological innovations, and collaborate with technology researchers, and embrace multidisciplinary intersections for improved healthcare.

## Author contributions

ZW: Conceptualization, Writing – original draft. NZ: Conceptualization, Writing – original draft. PL: Supervision, Writing – review & editing. YX: Supervision, Writing – review & editing. NY: Funding acquisition, Supervision, Writing – review & editing.

## References

[1] Teo ZL Tham YC Yu M Chee ML Rim TH Cheung N. Global prevalence of diabetic retinopathy and projection of Burden through 2045: Systematic review and meta-analysis. Ophthalmology (2021) 128:1580–91. doi: 10.1016/j.ophtha.2021.04.027 33940045

[2] GBD. Blindness and Vision Impairment Collaborators; Vision Loss Expert Group of the Global Burden of Disease Study. Causes of blindness and vision impairment in 2020 and trends over 30 years, and prevalence of avoidable blindness in relation to VISION 2020: the Right to Sight: an analysis for the Global Burden of Disease Study. Lancet Glob Health (2019) 9(2):e144–60. doi: 10.1016/S2214-109X(20)30489-7 PMC7820391 33275949

[3] Flaxman SR Bourne RRA Resnikoff S Ackland P Braithwaite T Cicinelli MV. Global causes of blindness and distance vision impairment 1990–2020: A systematic review and meta-analysis. Lancet Glob Health (2017) 5:e1221–34. doi: 10.1016/S2214-109X(17)30393-5 29032195

[4] Orji A Rani PK Narayanan R Sahoo NK Das T. The economic burden of diabetic retinopathy care at a tertiary eye care center in South India. Indian J Ophthalmol (2021) 69:666–70. doi: 10.4103/ijo.IJO_1538_20 PMC7942062 33595498

[5] Wykoff CC. Impact of intravitreal pharmacotherapies including antivascular endothelial growth factor and corticosteroid agents on diabetic retinopathy. Curr Opin Ophthalmol (2017) 28:213–8. doi: 10.1097/ICU.0000000000000364 28376510

[6] Sugimoto M Ichio A Kondo M. Short pulse duration high-power laser photocoagulation during vitrectomy for diabetic retinopathy reduces postoperative inflammation. PloS One (2015) 10:e0135126. doi: 10.1371/journal.pone.0135126 26273824 PMC4537124

[7] Li JO Liu H Ting DSJ Jeon S Chan RVP Kim JE. Digital technology, tele-medicine and artificial intelligence in ophthalmology: A global perspective. Prog Retin Eye Res (2021) 82:100900. doi: 10.1016/j.preteyeres.2020.100900 32898686 PMC7474840

[8] Liu Y Wu N. Progress of nanotechnology in diabetic retinopathy treatment. Int J Nanomed (2021) 16:1391–403. doi: 10.2147/IJN.S294807 PMC7917322 33658779

[9] Silva M Peng T Zhao X Li S Farhan M Zheng W. Recent trends in drug-delivery systems for the treatment of diabetic retinopathy and associated fibrosis. Adv Drug Deliv Rev (2021) 173:439–60. doi: 10.1016/j.addr.2021.04.007 33857553

[10] Oshitari T. The pathogenesis and therapeutic approaches of diabetic neuropathy in the retina. Int J Mol Sci (2021) 22:9050. doi: 10.3390/ijms22169050 34445756 PMC8396448

[11] Hammes HP. Diabetic retinopathy: Hyperglycaemia, oxidative stress and beyond. Diabetologia (2018) 61:29–38. doi: 10.1007/s00125-017-4435-8 28942458

[12] Early Treatment Diabetic Retinopathy Study Research Group. Grading diabetic retinopathy from stereoscopic color fundus photographs – An extension of the modified Airlie House classification: ETDRS Report number 10. Ophthalmology (2020) 127:S99–119. doi: 10.1016/j.ophtha.2020.01.030 32200833

[13] Beltramo E Porta M. Pericyte loss in diabetic retinopathy: Mechanisms and consequences. Curr Med Chem (2013) 20:3218–25. doi: 10.2174/09298673113209990022 23745544

[14] Bianchi E Ripandelli G Taurone S Feher J Plateroti R Kovacs I. Age and diabetes related changes of the retinal capillaries: An ultrastructural and immunohistochemical study. Int J Immunopathol Pharmacol (2016) 29:40–53. doi: 10.1177/0394632015615592 26604209 PMC5806738

[15] Liu C Ge HM Liu BH Dong R Shan K Chen X. Targeting pericyte-endothelial cell crosstalk by circular RNA-cPWWP2A inhibition aggravates diabetes-induced microvascular dysfunction. Proc Natl Acad Sci USA (2019) 116:7455–64. doi: 10.1073/pnas.1814874116 PMC6462073 30914462

[16] Gupta N Mansoor S Sharma A Sapkal A Sheth J Falatoonzadeh P. Diabetic retinopathy and VEGF. Open Ophthalmol J (2013) 7:4–10. doi: 10.2174/1874364101307010004 23459241 PMC3580758

[17] Sohn EH van Dijk HW Jiao C Kok PH Jeong W Demirkaya N. Retinal neurodegeneration may precede microvascular changes characteristic of diabetic retinopathy in diabetes mellitus. Proc Natl Acad Sci USA (2016) 113:E2655–64. doi: 10.1073/pnas.1522014113 PMC4868487 27114552

[18] Boss JD Singh PK Pandya HK Tosi J Kim C Tewari A. Assessment of neurotrophins and inflammatory mediators in vitreous of patients with diabetic retinopathy. Invest. Ophthalmol Vis Sci (2017) 58:5594–603. doi: 10.1167/iovs.17-21973 PMC5667399 29084332

[19] Altmann C Schmidt MHH. The role of microglia in diabetic retinopathy: Inflammation, microvasculature defects and neurodegeneration. Int J Mol Sci (2018) 19:110. doi: 10.3390/ijms19010110 29301251 PMC5796059

[20] Forrester JV Kuffova L Delibegovic M. The role of inflammation in diabetic retinopathy. Front Immunol (2020) 11:583687. doi: 10.3389/fimmu.2020.583687 33240272 PMC7677305

[21] Oshitari T Yoshida-Hata N Yamamoto S. Effect of neurotrophin-4 on endoplasmic reticulum stress-related neuronal apoptosis in diabetic and high glucose exposed rat retinas. Neurosci Lett (2011) 501:102–6. doi: 10.1016/j.neulet.2011.06.057 21767604

[22] Wang CF Yuan JR Qin D Gu JF Zhao BJ Zhang L. Protection of tauroursodeoxycholic acid on high glucose-induced human retinal microvascular endothelial cells dysfunction and streptozotocin-induced diabetic retinopathy rats. J Ethnopharmacol (2016) 185:162–70. doi: 10.1016/j.jep.2016.03.026 26988565

[23] Davinelli S Chiosi F Di Marco R Costagliola C Scapagnini G. Cytoprotective effects of citicoline and homotaurine against glutamate and high glucose neurotoxicity in primary cultured retinal cells. Oxid Med Cell Longev (2017) 2017:2825703. doi: 10.1155/2017/2825703 29163753 PMC5661090

[24] Suzumura A Kaneko H Funahashi Y Takayama K Nagaya M Ito S. n-3 fatty acid and its metabolite 18-hepe ameliorate retinal neuronal cell dysfunction by enhancing Müller BDNF in diabetic retinopathy. Diabetes (2020) 69:724–35. doi: 10.2337/db19-0550 32029482

[25] Cai C Meng C He S Gu C Lhamo T Draga D. DNA methylation in diabetic retinopathy: pathogenetic role and potential therapeutic targets. Cell Biosci (2022) 12(1):186. doi: 10.1186/s13578-022-00927-y 36397159 PMC9673308

[26] Wang W Lo ACY. Diabetic retinopathy: Pathophysiology and treatments. Int J Mol Sci (2018) 19:1816. doi: 10.3390/ijms19061816 29925789 PMC6032159

[27] Yang S Guo X Cheng W Seth I Bulloch G Chen Y. Genome-wide DNA methylation analysis of extreme phenotypes in the identification of novel epigenetic modifications in diabetic retinopathy. Clin Epigenet (2022) 14(1):137. doi: 10.1186/s13148-022-01354-z PMC9623976 36316758

[28] Du X Yang L Kong L Sun Y Shen K Cai Y. Metabolomics of various samples advancing biomarker discovery and pathogenesis elucidation for diabetic retinopathy. Front Endocrinol (2022) 13:1037164. doi: 10.3389/fendo.2022.1037164 PMC9646596 36387907

[29] Li H Liu X Zhong H Fang J Li X Shi R. Research progress on the pathogenesis of diabetic retinopathy. BMC Ophthalmol (2023) 23(1):372. doi: 10.1186/s12886-023-03118-6 37697295 PMC10494348

[30] Yue T Shi Y Luo S Weng J Wu Y Zheng X. The role of inflammation in immune system of diabetic retinopathy: Molecular mechanisms, pathogenetic role and therapeutic implications. Front Immunol (2022) 13:1055087. doi: 10.3389/fimmu.2022.1055087 36582230 PMC9792618

[31] Gozawa M Takamura Y Miyake S Matsumura T Morioka M Yamada Y. Photocoagulation of the retinal nonperfusion area prevents the expression of the vascular endothelial growth factor in an animal model. Invest. Ophthalmol Vis Sci (2017) 58:5946–53. doi: 10.1167/iovs.17-22739 29098298

[32] Chew EY Ferris FL Csaky KG Murphy RP Agrón E Thompson DJ. The long-term effects of laser photocoagulation treatment in patients with diabetic retinopathy: The early treatment diabetic retinopathy follow-up study. Ophthalmology (2003) 110:1683–9. doi: 10.1016/S0161-6420(03)00579-7 13129862

[33] Tsilimbaris MK Kontadakis GA Tsika C Papageorgiou D Charoniti M. Effect of panretinal photocoagulation treatment on vision-related quality of life of patients with proliferative diabetic retinopathy. Retina (2013) 33:756–61. doi: 10.1097/IAE.0b013e31826b0c06 23190918

[34] Blumenkranz MS Yellachich D Andersen DE Wiltberger MW Mordaunt D Marcellino GR. Semiautomated patterned scanning laser for retinal photocoagulation. Retina (2006) 26:370–6. doi: 10.1097/00006982-200603000-00024 16508446

[35] Paulus YM Kaur K Egbert PR Blumenkranz MS Moshfeghi DM. Human histopathology of Pascal laser burns. Eye (Lond) (2013) 27:995–6. doi: 10.1038/eye.2013.100 PMC3740304 23722723

[36] Inan S Polat O Yıgıt S Inan UU. Pascal laser platform produces less pain responses compared to conventional laser system during the panretinal photocoagulation: A randomized clinical trial. Afr Health Sci (2018) 18:1010–7. doi: 10.4314/ahs.v18i4.22 PMC6354857 30766567

[37] Nagpal M Marlecha S Nagpal K. Comparison of laser photocoagulation for diabetic retinopathy using 532-nm standard laser versus multispot pattern scan laser. Retina (2010) 30:452–8. doi: 10.1097/IAE.0b013e3181c70127 20216293

[38] Seifert E Tode J Pielen A Theisen-Kunde D Framme C Roider J. Selective retina therapy: Toward an optically controlled automatic dosing. J Biomed Opt (2018) 23:1–12. doi: 10.1117/1.JBO.23.11.115002 30392199

[39] Brader HS Young LH. Subthreshold diode micropulse laser: A review. Semin Ophthalmol (2016) 31:30–9. doi: 10.3109/08820538.2015.1114837 26959127

[40] Moisseiev E Abbassi S Thinda S Yoon J Yiu G Morse LS. Subthreshold micropulse laser reduces anti-VEGF injection burden in patients with diabetic macular edema. Eur J Ophthalmol (2018) 28:68–73. doi: 10.5301/ejo.5001000 28731494

[41] Lai K Zhao H Zhou L Huang C Zhong X Gong Y. Subthreshold pan-retinal photocoagulation using endpoint management algorithm for severe nonproliferative diabetic retinopathy: A paired controlled pilot prospective study. Ophthal Res (2021) 64:648–55. doi: 10.1159/000512296 33053561

[42] Kozak I Oster SF Cortes MA Dowell D Hartmann K Kim JS. Clinical evaluation and treatment accuracy in diabetic macular edema using navigated laser photocoagulator NAVILAS. Ophthalmology (2011) 118:1119–24. doi: 10.1016/j.ophtha.2010.10.007 21269701

[43] Paulus YM Qin Y Yu Y Fu J Wang X Yang X. Photo-mediated ultrasound therapy to treat retinal neovascularization. Annu Int Conf IEEE Eng. Med Biol Soc. Annu Int Conf IEEE Eng. Med Biol Soc (2020) 2020:5244–7. doi: 10.1109/EMBC44109.2020.9175882 PMC9728478 33019167

[44] Lee GH Jeon C Mok JW Shin S Kim SK Han HH. Smart wireless near-infrared light emitting contact lens for the treatment of diabetic retinopathy. Adv Sci (Weinh) (2022) 9(9):e2103254. doi: 10.1002/advs.202103254 35092362 PMC8948592

[45] Li J Paulus YM. Advances in retinal laser therapy. Int J ophthalmic Res (2018) 4(1):259–64. doi: 10.17554/j.issn.2409-5680.2018.04.70 PMC6824197 31681861

[46] Everett LA Paulus YM. Laser therapy in the treatment of diabetic retinopathy and diabetic macular edema. Curr Diabetes Rep (2021) 21(9):35. doi: 10.1007/s11892-021-01403-6 PMC8420141 34487257

[47] Yang KB Feng H Zhang H. Effects of the COVID-19 pandemic on anti-vascular endothelial growth factor treatment in China. Front Med (Lausanne) (2020) 7:576275. doi: 10.3389/fmed.2020.576275 33381511 PMC7768079

[48] Yokota R Inoue M Itoh Y Rii T Hirota K Hirakata A. Comparison of microinsicion vitrectomy and conventional 20-gauge vitrectomy for severe proliferative diabetic retinopathy. Jpn J Ophthalmol (2015) 59:288–94. doi: 10.1007/s10384-015-0396-y 26202442

[49] Charles S Ho AC Dugel PU Riemann CD Berrocal MH Gupta S. Clinical comparison of 27-gauge and 23-gauge instruments on the outcomes of pars plana vitrectomy surgery for the treatment of vitreoretinal diseases. Curr Opin Ophthalmol (2020) 31:185–91. doi: 10.1097/ICU.0000000000000659 32235251

[50] Tomita Y Kurihara T Uchida A Nagai N Shinoda H Tsubota K. Wide-angle viewing system versus conventional indirect ophthalmoscopy for scleral buckling. Sci Rep (2015) 5:13256. doi: 10.1038/srep13256 26329974 PMC4557079

[51] Zhao XY Xia S Chen YX. Antivascular endothelial growth factor agents pretreatment before vitrectomy for complicated proliferative diabetic retinopathy: A meta-analysis of randomised controlled trials. Br J Ophthalmol (2018) 102:1077–85. doi: 10.1136/bjophthalmol-2017-311344 PMC6059039 29246890

[52] Nakashima H Iwama Y Tanioka K Emi K. Paracentral acute middle maculopathy following vitrectomy for proliferative diabetic retinopathy: Incidence, risk factors, and clinical characteristics. Ophthalmology (2018) 125:1929–36. doi: 10.1016/j.ophtha.2018.07.006 30126649

[53] Chen G Tzekov R Li W Jiang F Mao S Tong Y. INCIDENCE OF endophthalmitis after vitrectomy: A systematic review and meta-analysis. Retina (2019) 39:844–52. doi: 10.1097/IAE.0000000000002055 29370034

[54] Sun D Lin Y Zeng R Yang Z Deng X Lan Y. The incidence and risk factors of neovascular glaucoma secondary to proliferative diabetic retinopathy after vitrectomy. Eur J Ophthalmol (2021) 31:3057–67. doi: 10.1177/1120672120980686 33334171

[55] Penn JS Madan A Caldwell RB Bartoli M Caldwell RW Hartnett ME. Vascular endothelial growth factor in eye disease. Prog Retin Eye Res (2008) 27:331–71. doi: 10.1016/j.preteyeres.2008.05.001 PMC3682685 18653375

[56] Holmes DI Zachary I. The vascular endothelial growth factor (VEGF) family: Angiogenic factors in health and disease. Genome Biol (2005) 6:209. doi: 10.1186/gb-2005-6-2-209 15693956 PMC551528

[57] Miller JW Le Couter J Strauss EC Ferrara N. Vascular endothelial growth factor A in intraocular vascular disease. Ophthalmology (2013) 120:106–14. doi: 10.1016/j.ophtha.2012.07.038 23031671

[58] Papadopoulos N Martin J Ruan Q Rafique A Rosconi MP Shi E. Binding and neutralization of vascular endothelial growth factor (VEGF) and related ligands by VEGF Trap, ranibizumab and bevacizumab. Angiogenesis (2012) 15:171–85. doi: 10.1007/s10456-011-9249-6 PMC3338918 22302382

[59] Park YJ Ahn J Kim TW Park SJ Joo K Park KH. Efficacy of bevacizumab for vitreous haemorrhage in proliferative diabetic retinopathy with prior complete panretinal photocoagulation. Eye (Lond) (2021) 35:3056–63. doi: 10.1038/s41433-020-01384-y PMC8526678 33420422

[60] Ip MS Domalpally A Sun JK Ehrlich JS. Long-term effects of therapy with ranibizumab on diabetic retinopathy severity and baseline risk factors for worsening retinopathy. Ophthalmology (2015) 122:367–74. doi: 10.1016/j.ophtha.2014.08.048 25439595

[61] Gross JG Glassman AR Liu D Sun JK Antoszyk AN Baker CW. Five-year outcomes of panretinal photocoagulation vs intravitreous ranibizumab for proliferative diabetic retinopathy: A randomized clinical trial. JAMA Ophthalmol (2018) 136:1138–48. doi: 10.1001/jamaophthalmol.2018.3255 PMC6233839 30043039

[62] Tamura R Tanaka T Miyake K Yoshida K Sasaki H. Bevacizumab for Malignant gliomas: Current indications, mechanisms of action and resistance, and markers of response. Brain Tumor Pathol (2017) 34:62–77. doi: 10.1007/s10014-017-0284-x 28386777

[63] Platania CB Di Paola L Leggio GM Romano GL Drago F Salomone S. Molecular features of interaction between VEGFA and anti-angiogenic drugs used in retinal diseases: A computational approach. Front Pharmacol (2015) 6:248. doi: 10.3389/fphar.2015.00248 26578958 PMC4624855

[64] Nicolò M Ferro Desideri L Vagge A Traverso CE. Faricimab: An investigational agent targeting the Tie-2/angiopoietin pathway and VEGF-A for the treatment of retinal diseases. Expert Opin Investig Drugs (2021) 30:193–200. doi: 10.1080/13543784.2021.1879791 33471572

[65] Tadayoni R Sararols L Weissgerber G Verma R Clemens A Holz FG. Brolucizumab: A newly developed anti-VEGF molecule for the treatment of neovascular age-related macular degeneration. Ophthalmologica (2021) 244:93–101. doi: 10.1159/000513048 33197916

[66] Iglicki M González DP Loewenstein A Zur D. Next-generation anti-VEGF agents for diabetic macular oedema. Eye (Lond) (2022) 36:273–7. doi: 10.1038/s41433-021-01722-8 PMC8807622 34373607

[67] Rosenfeld PJ Brown DM Heier JS Boyer DS Kaiser PK Chung CY. Ranibizumab for neovascular age-related macular degeneration. N Engl J Med (2006) 355(14):1419–31. doi: 10.1056/NEJMoa054481 17021318

[68] Busbee BG Ho AC Brown DM Heier JS Suñer IJ Li Z. Twelve-month efficacy and safety of 0.5 mg or 2.0 mg ranibizumab in patients with subfoveal neovascular age-related macular degeneration. Ophthalmology (2013) 120(5):1046–56. doi: 10.1016/j.ophtha.2012.10.014 23352196

[69] Sepah YJ Sadiq MA Boyer D Callanan D Gallemore R Bennett M. Twenty-four-month outcomes of the ranibizumab for edema of the macula in diabetes - protocol 3 with high dose (READ-3) study. Ophthalmology (2016) 123(12):2581–7. doi: 10.1016/j.ophtha.2016.08.040 27707550

[70] Do DV Sepah YJ Boyer D Callanan D Gallemore R Bennett M. Month-6 primary outcomes of the READ-3 study (Ranibizumab for Edema of the mAcula in Diabetes-Protocol 3 with high dose). Eye (London England) (2015) 29(12):1538–44. doi: 10.1038/eye.2015.142 PMC5129796 26228291

[71] Kellner U Bedar MS Weinitz S Farmand G Sürül EN Weide SM. Treatment contentment and preference of patients undergoing intravitreal anti-VEGF therapy. Graefe's Arch Clin Exp Ophthalmol = Albrecht von Graefes Archiv fur klinische und experimentelle Ophthalmologie (2021) 259(12):3649–54. doi: 10.1007/s00417-021-05324-8 PMC8298185 34296345

[72] Obeid A Su D Patel SN Uhr JH Borkar D Gao X. Outcomes of eyes lost to follow-up with proliferative diabetic retinopathy that received panretinal photocoagulation versus intravitreal anti-vascular endothelial growth factor. Ophthalmology (2019) 126:407–13. doi: 10.1016/j.ophtha.2018.07.027 30077614

[73] Dugel PU Koh A Ogura Y Jaffe GJ Schmidt-Erfurth U Brown DM. HAWK and HARRIER: Phase 3, multicenter, randomized, double-masked trials of brolucizumab for neovascular age-related macular degeneration. Ophthalmology (2020) 127:72–84. doi: 10.1016/j.ophtha.2019.04.017 30986442

[74] Bhattacharya D Chaudhuri S Singh MK Chaudhuri S. T11TS inhibits angiopoietin-1/Tie-2 signaling, EGFR activation and Raf/MEK/ERK pathway in brain endothelial cells restraining angiogenesis in glioma model. Exp Mol Pathol (2015) 98:455–66. doi: 10.1016/j.yexmp.2015.03.026 25797371

[75] Campochiaro PA Khanani A Singer M Patel S Boyer D Dugel P. Enhanced benefit in diabetic macular edema from AKB-9778 Tie2 activation combined with vascular endothelial growth factor suppression. Ophthalmology (2016) 123:1722–30. doi: 10.1016/j.ophtha.2016.04.025 27236272

[76] Yang SF Su YC Lim CC Huang JY Hsu SM Wu LW. Risk of dialysis in patients receiving intravitreal anti-vascular endothelial growth factor treatment: a population-based cohort study. Aging (2022) 14(12):5116–30. doi: 10.18632/aging.204133 PMC9271293 35724264

[77] Lee IT Corona ST Wong TP Flynn HWJR Wykoff CC. Favorable anti-VEGF crunch syndrome: Nonsurgical relief of vitreoretinal traction in eyes with proliferative diabetic retinopathy and tractional retinal detachment. Ophthalmic surgery lasers Imaging retina (2022) 53(8):455–9. doi: 10.3928/23258160-20220628-01 35951712

[78] Striglia E Caccioppo A Castellino N Reibaldi M Porta M. Emerging drugs for the treatment of diabetic retinopathy. Expert Opin Emerging Drugs (2020) 25(3):261–71. doi: 10.1080/14728214.2020.1801631 32715794

[79] Hackett SF Wiegand S Yancopoulos G Campochiaro PA. Angiopoietin-2 plays an important role in retinal angiogenesis. J Cell Physiol (2002) 192(2):182–7. doi: 10.1002/jcp.10128 12115724

[80] Feng Y vom Hagen F Pfister F Djokic S Hoffmann S Back W. Impaired pericyte recruitment and abnormal retinal angiogenesis as a result of angiopoietin-2 overexpression. Thromb Haemostasis (2007) 97(1):99–108. doi: 10.1160/TH06-05-0277 17200776

[81] Joussen AM Ricci F Paris LP Korn C Quezada-Ruiz C Zarbin M. Angiopoietin/Tie2 signalling and its role in retinal and choroidal vascular diseases: a review of preclinical data. Eye (London England) (2021) 35(5):1305–16. doi: 10.1038/s41433-020-01377-x PMC8182896 33564135

[82] Liberski S Wichrowska M Kocięcki J. Aflibercept versus faricimab in the treatment of neovascular age-related macular degeneration and diabetic macular edema: A review. Int J Mol Sci (2022) 23(16):9424. doi: 10.3390/ijms23169424 36012690 PMC9409486

[83] Sahni J Patel SS Dugel PU Khanani AM Jhaveri CD Wykoff CC. Simultaneous inhibition of angiopoietin-2 and vascular endothelial growth factor-A with faricimab in diabetic macular edema: BOULEVARD phase 2 randomized trial. Ophthalmology (2019) 126(8):1155–70. doi: 10.1016/j.ophtha.2019.03.023 30905643

[84] Van Geest RJ Lesnik-Oberstein SY Tan HS Mura M Goldschmeding R Van Noorden CJ. A shift in the balance of vascular endothelial growth factor and connective tissue growth factor by bevacizumab causes the angiofibrotic switch in proliferative diabetic retinopathy. Br J Ophthalmol (2012) 96:587–90. doi: 10.1136/bjophthalmol-2011-301005 PMC3308470 22289291

[85] Wykoff CC Abreu F Adamis AP Basu K Eichenbaum DA Haskova Z. Efficacy, durability, and safety of intravitreal faricimab with extended dosing up to every 16 weeks in patients with diabetic macular oedema (YOSEMITE and RHINE): two randomised, double-masked, phase 3 trials. Lancet (London England) (2022) 399(10326):741–55. doi: 10.1016/S0140-6736(22)00018-6 35085503

[86] Watkins C Paulo T Bührer C Holekamp NM Bagijn M. Comparative efficacy, durability and safety of faricimab in the treatment of diabetic macular edema: A systematic literature review and network meta-analysis. Adv Ther (2023) 40(12):5204–21. doi: 10.1007/s12325-023-02675-y PMC10937806 37751021

[87] Kuiper EJ Van Nieuwenhoven FA de Smet MD van Meurs JC Tanck MW Oliver N. The angio-fibrotic switch of VEGF and CTGF in proliferative diabetic retinopathy. PloS One (2008) 3:e2675. doi: 10.1371/journal.pone.0002675 18628999 PMC2443281

[88] Klaassen I van Geest RJ Kuiper EJ van Noorden CJ Schlingemann RO. The role of CTGF in diabetic retinopathy. Exp Eye Res (2015) 133:37–48. doi: 10.1016/j.exer.2014.10.016 25819453

[89] Storey PP Obeid A Pancholy M Goodman J Borkar D Su D. Ocular hypertension after intravitreal injection of 2-Mg triamcinolone. Retina (2020) 40:75–9. doi: 10.1097/IAE.0000000000002361 30308561

[90] Liao M Huang Y Wang J Meng X Liu Y Yu J. Long-term outcomes of administration of intravitreal triamcinolone acetonide after posterior vitreous detachment during pars plana vitrectomy for proliferative diabetic retinopathy. Br J Ophthalmol (2023) 107:560–4. doi: 10.1136/bjophthalmol-2021-320332 34844917

[91] Hu L Chen Q Du Z Wang W Zhao G. Evaluation of vitrectomy combined preoperative intravitreal ranibizumab and postoperative intravitreal triamcinolone acetonide for proliferative diabetic retinopathy. Int Ophthalmol (2021) 41:1635–42. doi: 10.1007/s10792-021-01703-6 33538931

[92] Xu K Qian H Zou M. Triamcinolone acetonide combined with aminoguanidine inhibits inflammation and oxidative stress, improves vascular endothelial and retinal function and reduces VEGF expression in diabetic retinopathy patients. Exp Ther Med (2020) 19:2519–26. doi: 10.3892/etm.2020.8478 PMC7086164 32256730

[93] Kambhampati SP Mishra MK Mastorakos P Oh Y Lutty GA Kannan RM. Intracellular delivery of dendrimer triamcinolone acetonide conjugates into microglial and human retinal pigment epithelial cells. Eur J Pharm Biopharm (2015) 95:239–49. doi: 10.1016/j.ejpb.2015.02.013 PMC4861086 25701805

[94] Cho H Kambhampati SP Lai MJ Zhou L Lee G Xie Y. Dendrimer-triamcinolone acetonide reduces neuroinflammation, pathological angiogenesis, and neuroretinal dysfunction in ischemic retinopathy. Adv Ther (Weinh) (2021) 4:2000181. doi: 10.1002/adtp.202000181 34527806 PMC8436818

[95] Crespo-Garcia S Tsuruda PR Dejda A Ryan RD Fournier F Chaney SY. Pathological angiogenesis in retinopathy engages cellular senescence and is amenable to therapeutic elimination *via* BCL-xL inhibition. Cell Metab (2021) 33:818–832.e7. doi: 10.1016/j.cmet.2021.01.011 33548171

[96] Coppé JP Patil CK Rodier F Sun Y Muñoz DP Goldstein J. Senescence-associated secretory phenotypes reveal cell-nonautonomous functions of oncogenic RAS and the p53 tumor suppressor. PloS Biol (2008) 6:2853–68. doi: 10.1371/journal.pbio.0060301 PMC2592359 19053174

[97] Hassan JW Bhatwadekar AD. Senolytics in the treatment of diabetic retinopathy. Front Pharmacol (2022) 13:896907. doi: 10.3389/fphar.2022.896907 36091769 PMC9462063

[98] Tsuruda P Chaney S Dejda A Dasgupta S Crespo-Garcia S Rao S. UBX1325, a small molecule inhibitor of Bcl-xL, attenuates vascular dysfunction in two animal models of retinopathy. Invest Ophthalmol Vis Sci (2021) 62(8):1163.

[99] Sreekumar PG Hinton DR Kannan R. The emerging role of senescence in ocular disease. Oxid Med Cell Longev (2020) 2020:2583601. doi: 10.1155/2020/2583601 32215170 PMC7085400

[100] Ogrodnik M Miwa S Tchkonia T Tiniakos D Wilson CL Lahat A. Cellular senescence drives age-dependent hepatic steatosis. Nat Commun (2017) 8:15691. doi: 10.1038/ncomms15691 28608850 PMC5474745

[101] Kim SR Jiang K Ogrodnik M Chen X Zhu XY Lohmeier H. Increased renal cellular senescence in murine high-fat diet: effect of the senolytic drug quercetin. Trans Res J Lab Clin Med (2019) 213:112–23. doi: 10.1016/j.trsl.2019.07.005 PMC6783353 31356770

[102] Di Micco R Krizhanovsky V Baker D d'Adda di Fagagna F. Cellular senescence in ageing: from mechanisms to therapeutic opportunities. Nat Rev Mol Cell Biol (2021) 22(2):75–95. doi: 10.1038/s41580-020-00314-w 33328614 PMC8344376

[103] Kang Q Yang C. Oxidative stress and diabetic retinopathy: Molecular mechanisms, pathogenetic role and therapeutic implications. Redox Biol (2020) 37:101799. doi: 10.1016/j.redox.2020.101799 33248932 PMC7767789

[104] Rodríguez ML Pérez S Mena-Mollá S Desco MC Ortega Á.L. Oxidative stress and microvascular alterations in diabetic retinopathy: Future therapies. Oxid Med Cell Longev (2019) 2019:4940825. doi: 10.1155/2019/4940825 31814880 PMC6878793

[105] Wei Y Gong J Xu Z Duh EJ. Nrf2 promotes reparative angiogenesis through regulation of NADPH oxidase-2 in oxygen-induced retinopathy. Free Radic Biol Med (2016) 99:234–43. doi: 10.1016/j.freeradbiomed.2016.08.013 PMC8565612 27521459

[106] Du J Wang Y Tu Y Guo Y Sun X Xu X. A prodrug of epigallocatechin-3-gallate alleviates high glucose-induced pro-angiogenic factor production by inhibiting the ROS/TXNIP/NLRP3 inflammasome axis in retinal Muller cells. Exp Eye Res (2020) 196:108065. doi: 10.1016/j.exer.2020.108065 32407725

[107] Wang L Sun X Zhu M Du J Xu J Qin X. Epigallocatechin-3-gallate stimulates autophagy and reduces apoptosis levels in retinal Muller cells under high-glucose conditions. Exp Cell Res (2019) 380:149–58. doi: 10.1016/j.yexcr.2019.04.014 30998948

[108] Xie T Chen X Chen W Huang S Peng X Tian L. Curcumin is a potential adjuvant to alleviates diabetic retinal injury *via* reducing oxidative stress and maintaining Nrf2 pathway homeostasis. Front Pharmacol (2021) 12:796565. doi: 10.3389/fphar.2021.796565 34955862 PMC8702852

[109] Li J Yu S Ying J Shi T Wang P. Resveratrol prevents ROS-induced apoptosis in high glucose-treated retinal capillary endothelial cells *via* the activation of AMPK/Sirt1/PGC-1alpha pathway. Oxid Med Cell Longev (2017) 2017:7584691. doi: 10.1155/2017/7584691 29213353 PMC5682085

[110] Zeng K Wang Y Yang N Wang D Li S Ming J. Resveratrol inhibits diabetic-induced Muller cells apoptosis through MicroRNA-29b/Specificity Protein 1 pathway. Mol Neurobiol (2017) 54:4000–14. doi: 10.1007/s12035-016-9972-5 27311771

[111] Gong X Draper CS Allison GS Marisiddaiah R Rubin LP. Effects of the macular carotenoid lutein in human retinal pigment epithelial cells. Antioxidants (Basel) (2017) 6:100. doi: 10.3390/antiox6040100 29207534 PMC5745510

[112] Toragall V Baskaran V. Chitosan-sodium alginate-fatty acid nanocarrier system: lutein bioavailability, absorption pharmacokinetics in diabetic rat and protection of retinal cells against H2O2 induced oxidative stress *in vitro* . Carbohydr Polym (2021) 254:117409. doi: 10.1016/j.carbpol.2020.117409 33357895

[113] Shi C Wang P Airen S Brown C Liu Z Townsend JH. Nutritional and medical food therapies for diabetic retinopathy. Eye Vis (Lond) (2020) 7:33. doi: 10.1186/s40662-020-00199-y 32582807 PMC7310218

[114] Thakur S Gupta SK Ali V Singh P Verma M. Aldose reductase: A cause and a potential target for the treatment of diabetic complications. Arch Pharm Res (2021) 44:655–67. doi: 10.1007/s12272-021-01343-5 34279787

[115] Tammali R Reddy AB Srivastava SK Ramana KV. Inhibition of aldose reductase prevents angiogenesis in *vitro* and in *vivo* . Angiogenesis (2011) 14:209–21. doi: 10.1007/s10456-011-9206-4 PMC3103619 21409599

[116] Liu F Ma Y Xu Y. Taxifolin shows anticataractogenesis and attenuates diabetic retinopathy in STZ-diabetic rats *via* suppression of aldose reductase, oxidative stress, and MAPK signaling pathway. Endocr. Metab Immune Disord Drug Targets (2020) 20:599–608. doi: 10.2174/1871530319666191018122821 31656158

[117] Toyoda F Tanaka Y Ota A Shimmura M Kinoshita N Takano H. Effect of ranirestat, a new aldose reductase inhibitor, on diabetic retinopathy in SDT rats. J Diabetes Res (2014) 2014:672590. doi: 10.1155/2014/672590 25215304 PMC4158328

[118] Fabian E Reglodi D Horvath G Opper B Toth G Fazakas C. Pituitary adenylate cyclase activating polypeptide acts against neovascularization in retinal pigment epithelial cells. Ann N Y Acad Sci (2019) 1455:160–72. doi: 10.1111/nyas.14189 31317557

[119] Ola MS Nawaz MI El-Asrar AA Abouammoh M Alhomida AS. Reduced levels of brain derived neurotrophic factor (BDNF) in the serum of diabetic retinopathy patients and in the retina of diabetic rats. Cell Mol Neurobiol (2013) 33:359–67. doi: 10.1007/s10571-012-9901-8 PMC11498009 23271640

[120] Zerbini G Maestroni S Leocani L Mosca A Godi M Paleari R. Topical nerve growth factor prevents neurodegenerative and vascular stages of diabetic retinopathy. Front Pharmacol (2022) 13:1015522. doi: 10.3389/fphar.2022.1015522 36172176 PMC9510636

[121] Shao Y Chen J Dong LJ He X Cheng R Zhou K. A protective effect of PPARalpha in endothelial progenitor cells through regulating metabolism. Diabetes (2019) 68:2131–42. doi: 10.2337/db18-1278 PMC6804623 31451517

[122] Liu Q Zhang F Zhang X Cheng R Ma JX Yi J. Fenofibrate ameliorates diabetic retinopathy by modulating Nrf2 signaling and NLRP3 inflammasome activation. Mol Cell Biochem (2018) 445:105–15. doi: 10.1007/s11010-017-3256-x 29264825

[123] Tomita Y Lee D Miwa Y Jiang X Ohta M Tsubota K. Pemafibrate protects against retinal dysfunction in a murine model of diabetic retinopathy. Int J Mol Sci (2020) 21:6243. doi: 10.3390/ijms21176243 32872333 PMC7503472

[124] Takakura S Toyoshi T Hayashizaki Y Takasu T. Effect of ipragliflozin, an SGLT2 inhibitor, on progression of diabetic microvascular complications in spontaneously diabetic Torii fatty rats. Life Sci (2016) 147:125–31. doi: 10.1016/j.lfs.2016.01.042 26829386

[125] Gong Q Zhang R Wei F Fang J Zhang J Sun J. SGLT2 inhibitor-empagliflozin treatment ameliorates diabetic retinopathy manifestations and exerts protective effects associated with augmenting branched chain amino acids catabolism and transportation in db/db mice. Biomed Pharmacother (2022) 152:113222. doi: 10.1016/j.biopha.2022.113222 35671581

[126] Herat LY Matthews VB Rakoczy PE Carnagarin R Schlaich M. Focusing on sodium glucose Cotransporter-2 and the sympathetic nervous system: Potential impact in diabetic retinopathy. Int J Endocrinol (2018) 2018:9254126. doi: 10.1155/2018/9254126 30123269 PMC6079487

[127] Gong Q Xie J Li Y Liu Y Su G. Enhanced ROBO4 is mediated by up-regulation of HIF-1alpha/SP1 or reduction in miR-125b-5p/miR-146a-5p in diabetic retinopathy. J Cell Mol Med (2019) 23:4723–37. doi: 10.1111/jcmm.14369 PMC6584523 31094072

[128] Hu ML Edwards TL O’Hare F Hickey DG Wang JH Liu Z. Gene therapy for inherited retinal diseases: Progress and possibilities. Clin Exp Optom (2021) 104:444–54. doi: 10.1080/08164622.2021.1880863 33689657

[129] Wang JH Roberts GE Liu GS. Updates on gene therapy for diabetic retinopathy. Curr Diab Rep (2020) 20:22. doi: 10.1007/s11892-020-01308-w 32415508 PMC7228867

[130] Zhang X Das SK Passi SF Uehara H Bohner A Chen M. AAV2 delivery of Flt23k intraceptors inhibits murine choroidal neovascularization. Mol Ther (2015) 23:226–34. doi: 10.1038/mt.2014.199 PMC4445610 25306972

[131] Rakoczy EP Magno AL Lai CM Pierce CM Degli-Esposti MA Blumenkranz MS. Three-year follow-up of phase 1 and 2a rAAV. sFLT-1 subretinal gene therapy trials for exudative age-related macular degeneration. Am J Ophthalmol (2019) 204:113–23. doi: 10.1016/j.ajo.2019.03.006 30878487

[132] Tu L Wang JH Barathi VA Prea SM He Z Lee JH. AAV-mediated gene delivery of the calreticulin anti-angiogenic domain inhibits ocular neovascularization. Angiogenesis (2018) 21:95–109. doi: 10.1007/s10456-017-9591-4 29318471

[133] Jiang J Xia XB Xu HZ Xiong Y Song WT Xiong SQ. Inhibition of retinal neovascularization by gene transfer of small interfering RNA targeting HIF-1alpha and VEGF. J Cell Physiol (2009) 218:66–74. doi: 10.1002/jcp.21566 18767037

[134] Ao H Liu B Li H Lu L. Egr1 mediates retinal vascular dysfunction in diabetes mellitus *via* promoting p53 transcription. J Cell Mol Med (2019) 23:3345–56. doi: 10.1111/jcmm.14225 PMC6484413 30887692

[135] Gote V Sikder S Sicotte J Pal D. Ocular drug delivery: present innovations and future challenges. J Pharmacol Exp Ther (2019) 370(3):602–24. doi: 10.1124/jpet.119.256933 31072813

[136] Shatz W Aaronson J Yohe S Kelley RF Kalia YN. Strategies for modifying drug residence time and ocular bioavailability to decrease treatment frequency for back of the eye diseases. Expert Opin Drug Deliv (2019) 16:43–57. doi: 10.1080/17425247.2019.1553953 30488721

[137] Fangueiro JF Silva AM Garcia ML Souto EB. Current nanotechnology approaches for the treatment and management of diabetic retinopathy. Eur J Pharm Biopharm (2015) 95:307–22. doi: 10.1016/j.ejpb.2014.12.023 25536109

[138] Yan L Zhao B Liu X Li X Zeng C Shi H. Aligned nanofibers from polypyrrole/graphene as electrodes for regeneration of optic nerve *via* electrical stimulation. ACS Appl materials interfaces (2016) 8(11):6834–40. doi: 10.1021/acsami.5b12843 26926578

[139] Akbarzadeh A Rezaei-Sadabady R Davaran S Joo SW Zarghami N Hanifehpour Y. Liposome: Classification, preparation, and applications. Nanoscale Res Lett (2013) 8:102. doi: 10.1186/1556-276X-8-102 23432972 PMC3599573

[140] Tahara K Karasawa K Onodera R Takeuchi H. Feasibility of drug delivery to the eye’s posterior segment by topical instillation of PLGA nanoparticles. Asian J Pharm Sci (2017) 12:394–9. doi: 10.1016/j.ajps.2017.03.002 PMC7032217 32104351

[141] Sharma DS Wadhwa S Gulati M Kumar B Chitranshi N Gupta VK. Chitosan modified 5-fluorouracil nanostructured lipid carriers for treatment of diabetic retinopathy in rats: A new dimension to an anticancer drug. Int J Biol Macromol (2023) 224:810–30. doi: 10.1016/j.ijbiomac.2022.10.168 36302483

[142] Singh M Bharadwaj S Lee KE Kang SG. Therapeutic nanoemulsions in ophthalmic drug administration: Concept in formulations and characterization techniques for ocular drug delivery. J Control Release (2020) 328:895–916. doi: 10.1016/j.jconrel.2020.10.025 33069743

[143] Dave R Randhawa G Kim D Simpson M Hoare T. Microgels and nanogels for the delivery of poorly water-soluble drugs. Mol Pharm (2022) 19:1704–21. doi: 10.1021/acs.molpharmaceut.1c00967 35319212

[144] Yavuz B Bozdağ Pehlivan S Sümer Bolu B Nomak Sanyal R Vural İ Ünlü N. Dexamethasone – PAMAM dendrimer conjugates for retinal delivery: Preparation, characterization and in *vivo* evaluation. J Pharm Pharmacol (2016) 68:1010–20. doi: 10.1111/jphp.12587 27283886

[145] Andra VVSNL Pammi SVN Bhatraju LVKP Ruddaraju LK. A comprehensive review on novel liposomal methodologies, commercial formulations, clinical trials and patents. BioNanoScience (2022) 12:274–91. doi: 10.1007/s12668-022-00941-x PMC8790012 35096502

[146] Gonzalez-De la Rosa A Navarro-Partida J Altamirano-Vallejo JC Jauregui-Garcia GD Acosta-Gonzalez R Ibanez-Hernandez MA. Novel triamcinolone acetonide-loaded liposomal topical formulation improves contrast sensitivity outcome after femtosecond laser-assisted cataract surgery. J Ocul Pharmacol Ther (2019) 35:512–21. doi: 10.1089/jop.2019.0032 PMC6839423 31486694

[147] Bohley M Dillinger AE Braunger BM Tamm ER Goepferich A. Intravenous injection of cyclosporin A loaded lipid nanocapsules fights inflammation and immune system activation in a mouse model of diabetic retinopathy. Drug Deliv Transl Res (2023) 13(11):2807–18. doi: 10.1007/s13346-023-01350-7 PMC10545584 37208562

[148] Laddha UD Kshirsagar SJ. Formulation of PPAR-gamma agonist as surface modified PLGA nanoparticles for non-invasive treatment of diabetic retinopathy: *In vitro* and in *vivo* evidences. Heliyon (2020) 6:e04589. doi: 10.1016/j.heliyon.2020.e04589 32832706 PMC7432955

[149] Rong X Ji Y Zhu X Yang J Qian D Mo X. Neuroprotective effect of insulin-loaded chitosan nanoparticles/PLGA-PEG-PLGA hydrogel on diabetic retinopathy in rats. Int J Nanomed (2019) 14:45–55. doi: 10.2147/IJN.S184574 PMC6302824 30587984

[150] Jeong JH Nguyen HK Lee JE Suh W. Therapeutic effect of apatinib-loaded nanoparticles on diabetes-induced retinal vascular leakage. Int J Nanomed (2016) 11:3101–9. doi: 10.2147/IJN.S108452 PMC4940015 27462154

[151] Radwan SE El-Kamel A Zaki EI Burgalassi S Zucchetti E El-Moslemany RM. Hyaluronic-coated albumin nanoparticles for the non-invasive delivery of apatinib in diabetic retinopathy. Int J Nanomed (2021) 16:4481–94. doi: 10.2147/IJN.S316564 PMC8259843 34239300

[152] Amato R Giannaccini M Dal Monte M Cammalleri M Pini A Raffa V. Association of the somatostatin analog octreotide with magnetic nanoparticles for intraocular delivery: A possible approach for the treatment of diabetic retinopathy. Front Bioeng Biotechnol (2020) 8:144. doi: 10.3389/fbioe.2020.00144 32158755 PMC7051943

[153] Wang S Du S Wang W Zhang F. Therapeutic investigation of quercetin nanomedicine in a zebrafish model of diabetic retinopathy. BioMed Pharmacother (2020) 130:110573. doi: 10.1016/j.biopha.2020.110573 32745912

[154] Li YN Liang HW Zhang CL Qiu YM Wang D Wang HL. Ophthalmic solution of smart supramolecular peptides to capture semaphorin 4D against diabetic retinopathy. Advanced Sci (Weinheim Baden-Wurttemberg Germany) (2023) 10(3):e2203351. doi: 10.1002/advs.202203351 PMC9875641 36437109

[155] Ganugula R Arora M Dwivedi S Chandrashekar DS Varambally S Scott EM. Systemic anti-inflammatory therapy aided by curcumin-laden double-headed nanoparticles combined with injectable long-acting insulin in a rodent model of diabetes eye disease. ACS nano (2023) 17(7):6857–74. doi: 10.1021/acsnano.3c00535 36951721

[156] Lee JH Wang JH Chen J Li F Edwards TL Hewitt AW. Gene therapy for visual loss: Opportunities and concerns. Prog Retin Eye Res (2019) 68:31–53. doi: 10.1016/j.preteyeres.2018.08.003 30170104

